# Bioengineered Extracellular Vesicles in Emerging Cancer Vaccine Platforms

**DOI:** 10.1002/smsc.202500270

**Published:** 2025-09-04

**Authors:** Wonkyung Ahn, Yeram Lee, Gi‐Hoon Nam, Jae Bem You, Eun Jung Lee

**Affiliations:** ^1^ School of Chemical Engineering and Applied Chemistry Kyungpook National University Daegu 41566 Republic of Korea; ^2^ Department of Biochemistry & Molecular Biology Korea University College of Medicine Seoul 02842 Republic of Korea; ^3^ Department of Chemical Engineering and Applied Chemistry Chungnam National University Daejeon 34134 Republic of Korea

**Keywords:** cancer immunotherapies, cancer vaccines, dendritic cell‐derived extracellular vesicles, exosomes, extracellular vesicles, tumor‐derived extracellular vesicles, vaccines

## Abstract

Amid the evolving landscape of immunotherapy, the pursuit of safer, more precise, and broadly applicable vaccine platforms has intensified. While conventional technologies—such as lipid nanoparticle‐based mRNA systems—achieved unprecedented success in infectious disease prophylaxis, their limitations in safety and durability have prompted the search for alternatives to address more complex immunological challenges, particularly in oncology. Within this context, extracellular vesicle (EV)‐based vaccines have emerged as a next‐generation platform. These endogenously derived nanoscale vesicles, secreted by nearly all cell types, mirror the immunological identity of their origin and support diverse immune functions. Advances in EV research have enabled modular vaccine design through strategies such as antigen loading, surface engineering, and cytokine‐driven modulation. Depending on their cellular source—dendritic cells, macrophages, lymphocytes, or tumor cells—EVs exhibit distinct immunological properties that allow tailored engagement of immune responses. Rather than acting solely as delivery vehicles, they integrate antigen transport, immune activation, and adjuvant effects within a single structure. Recent progress in EV‐based cancer vaccine development is reviewed, encompassing vesicle biogenesis, engineering strategies, and delivery optimization, alongside emerging preclinical and clinical evidence supporting their translational potential. Finally, key challenges, including vesicle heterogeneity and manufacturing standardization, are outlined as factors that must be addressed to enable clinical advancement.

## Graphical abstract

This review highlights recent advances in engineering extracellular vesicles (EVs) as next‐generation cancer vaccines. It outlines their biological roles and antigen‐presenting capacity, explores key strategies for antigen loading and delivery optimization, and summarizes preclinical and clinical applications using EVs from diverse immune and tumor sources. The review concludes by addressing current challenges and future directions for clinical translation.

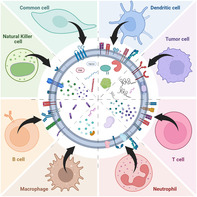

## 1 Introduction

Vaccination is among the most progressive and cost‐effective achievements in modern medicine, significantly reducing the global burden of infectious diseases. The unprecedented success of mRNA‐based vaccines during the COVID‐19 pandemic highlighted the potential of nucleic acid‐based platforms for rapid and scalable vaccine deployment. These innovations have not only accelerated prophylactic responses to emerging pathogens but also driven the rapid advancement of therapeutic vaccination strategies, including applications in cancer. Nonetheless, the widespread deployment of mRNA vaccines has also revealed critical limitations.^[^ ^1^ ^]^ Notably, many adverse effects have been attributed to synthetic lipid nanoparticles (LNPs) used as delivery carriers, raising concerns about their long‐term biocompatibility and safety.^[^ ^2^, ^3^, ^4^, ^5^ ^]^ Moreover, their intrinsic instability that necessitates stringent cold‐chain logistics^[^ ^6^, ^7^ ^]^ and their reliance on injectable delivery^[^ ^8^, ^9^ ^]^ continues to limit global accessibility. Despite notable advancements, current‐generation vaccine platforms—including LNP‐based mRNA technologies—face significant limitations, particularly in therapeutic applications. Such drawbacks underscore the urgent need for alternative platforms that can overcome these challenges. Consequently, various next‐generation vaccine platforms are actively being developed, such as self‐amplifying RNA (saRNA) systems,^[^ ^10^ ^]^ circular RNA (circRNA) constructs,^[^ ^11^ ^]^ virus‐like particles (VLPs),^[^ ^12^ ^]^ and synthetic polymer‐based nanocarriers.^[^ ^13^ ^]^

Among the emerging candidates, extracellular vesicle (EV)‐based vaccines have garnered considerable attention due to their natural origin, immunological flexibility, and engineering versatility.^[^ ^14^, ^15^ ^]^ EVs are cell‐secreted, lipid bilayer–enclosed nanoparticles ranging in size from ≈30 to 1000 nm, released by virtually all cell types through diverse biogenetic pathways.^[^ ^16^ ^]^ First observed in the mid‐20th century as membranous particles in blood plasma^[^ ^17^ ^]^ and later formalized as a unifying term^[^ ^18^ ^]^ to encompass secreted vesicles of diverse biogenetic origins,^[^ ^19^, ^20^, ^21^, ^22^ ^]^ EVs are now recognized as essential mediators of intercellular communication,^[^ ^23^, ^24^ ^]^ transporting bioactive proteins,^[^ ^25^ ^]^ RNAs,^[^ ^26^, ^27^ ^]^ and lipids^[^ ^28^ ^]^ under both physiological and pathological conditions. In accordance with evolving nomenclature guidelines, particularly those advocated by the International Society for Extracellular Vesicles (ISEV), the term “EVs” has been widely adopted as a generic descriptor encompassing subtypes such as exosomes, microvesicles, and other cell‐derived vesicles with overlapping physical and molecular characteristics.^[^ ^29^ ^]^ As inherently cell‐free entities, EVs offer significant therapeutic advantages, including enhanced safety, controllability, and scalability compared to living cell‐based therapies.^[^ ^30^, ^31^, ^32^ ^]^ Furthermore, their native membrane architecture, low immunogenicity, and inherent tissue tropism render them uniquely suited for vaccine delivery, particularly in contexts requiring precision, durability, and immunological balance.^[^ ^15^ ^]^ In addition, recent research has further highlighted the immunotherapeutic potential of EVs, particularly in oncology.^[^ ^33^ ^]^ Among various cellular sources, tumor‐derived EVs have been shown to contribute to immune evasion and metastatic dissemination, yet when properly engineered, they offer a valuable source of tumor‐associated antigen (TAA).^[^ ^34^, ^35^ ^]^ In contrast, dendritic cell (DC)‐derived EVs inherently express major histocompatibility complex (MHC) class I and II molecules, co‐stimulatory proteins, and immunomodulatory cytokines, enabling efficient priming of both CD8^+^ and CD4^+^ T cell responses.^[^ ^36^, ^37^ ^]^ Beyond these EV populations, EVs derived from macrophages,^[^ ^38^, ^39^ ^]^ B lymphocytes,^[^ ^40^, ^41^ ^]^ and T lymphocytes^[^ ^42^ ^]^ have also shown distinct immunostimulatory profiles, contributing to a growing interest in leveraging cell source‐dependent properties of EVs for both therapeutic and prophylactic vaccine applications across cancer and infectious diseases.^[^ ^43^, ^44^ ^]^

Beyond their immunological advantages, EVs offer notable practical benefits in terms of manufacturing and deployment.^[^ ^45^ ^]^ Among the various EV sources, DC‐derived EVs, as acellular biologics, represent the most clinically validated formulation, having been successfully produced under good manufacturing practice (GMP)‐compliant conditions.^[^ ^29^ ^]^ This precedent positions these EVs as a relatively scalable and standardizable model for future vaccine development, while EVs derived from other cellular sources may also hold untapped potential with respect to production feasibility with various opportunities for formulation versatility. Their excellent physicochemical stability further facilitate standardized processing and global distribution. Importantly, EVs can simultaneously function as delivery vehicles, immune activators, and antigen‐presenting entities—features that collectively overcome many limitations of current vaccine technologies.^[^ ^43^ ^]^

Building upon these insights, this review offers a comprehensive and critical appraisal of recent advances in EV‐based vaccine development, emphasizing their potential for oncological applications. We begin by examining the biological origin, structural characteristics, and immunological functions of EVs, establishing their role as natural, cell‐intrinsic carriers capable of antigen presentation and immune modulation. We then explore emerging bioengineering strategies designed to incorporate antigens in a desired form and improve immunogenicity and delivery precision, followed by an overview of state‐of‐the‐art EV characterization methods and mechanistic insights into their systemic delivery and biodistribution, as influenced by engineering strategies. Comparative evaluations of conventional vaccine systems are also included to contextualize the distinct advantages of EV‐based approaches. In addition, we summarize preclinical and clinical studies that leverage EVs derived from DCs, tumor cells, macrophages, and T lymphocytes—particularly in the development of cancer vaccines. Finally, we discuss remaining challenges and future directions for clinical translation, including large‐scale manufacturing, regulatory standardization, and therapeutic consistency. Through this review, we aim to facilitate the rational development of EV‐based vaccines as versatile and strategically important platforms for preventive and therapeutic immunization across diverse disease contexts.

## 2 Bioengineering Strategies Using EVs as Carrier for Antigen Delivery

Protein antigens represent the cornerstone of most modern vaccines, as they not only elicit protective immune responses against specific pathogens but also contribute to establishing durable immunological memory.^[^ ^46^ ^]^ Upon recognition by pattern recognition receptors (PRRs) expressed on innate immune cells, these antigens are internalized and processed by antigen‐presenting cells (APCs).^[^ ^47^ ^]^ This leads to the formation of peptide–MHC complexes, subsequently presented to T cells, initiating robust adaptive immune responses that engage both the cellular arm (CD8^+^ cytotoxic T lymphocytes (CTL)) and the humoral arm (CD4^+^ helper T cells and B cells). Thus, designing efficient and targeted antigen delivery systems is critical for enhancing vaccine potency and immunological specificity.^[^ ^48^ ^]^

In this section, we highlight current bioengineering strategies for utilizing EVs, versatile platforms for antigen delivery. These approaches encompass both endogenous and exogenous antigen‐loading techniques, along with advanced methods for enhancing innate immune recognition, high‐resolution characterization of engineered EVs, and elucidation of the systemic and molecular mechanisms governed by distinct delivery routes and engineering strategies. Collectively, these bioengineering approaches aim to exploit and enhance the intrinsic immunomodulatory potential of EVs for advanced vaccine development.

### 2.1 Defining EV Subtypes and their Functional Implications in Vaccine Design

Before discussing strategies for EV‐based vaccines, it is essential to clarify the current understanding of EV subtype classifications and their defining characteristics. Among these, “exosomes”—the smallest among EV subtypes (30–150 nm)—originate from the endosomal compartment of the cell and are released into the extracellular space upon fusion of multivesicular bodies (MVBs) with the plasma membrane.^[^ ^49^ ^]^ Meanwhile “ectosomes” (also called microvesicles or microparticles), ranging from 100–1000 nm, denote EVs directly released from the plasma membrane through cytoskeletal reorganization and outward membrane budding. Additional specialized subtypes include EVs produced during specific cellular processes, such as “migrasomes”^[^ ^50^, ^51^ ^]^ which emerge from retraction fibers during cell migration, and “apoptotic bodies”, which result from fragmentation of cells formed during programmed cell death; both generally exceed 500 nm in size.^[^ ^52^, ^53^ ^]^ Unfortunately, conventional EV isolation methods—such as differential ultracentrifugation, size‐exclusion chromatography (SEC), tangential flow filtration (TFF), and density‐gradient centrifugation—lack the specificity to purify vesicles according to their biogenetic origin, and no universal molecular markers exist to discriminate between subtypes such as exosomes and ectosomes, making definitive identification of EV subtypes challenging.^[^ ^54^ ^]^ In response to this limitation, a range of alternative strategies has emerged, including polymer precipitation,^[^ ^55^ ^]^ hybrid workflows such as SEC–TFF,^[^ ^56^ ^]^ and immunoaffinity‐based techniques.^[^ ^57^ ^]^ Among these, immunoaffinity‐based methods have received particular attention for their potential for selective enrichment of EVs based on surface‐expressed proteins or receptors.^[^ ^57^ ^]^ These methods offer practical advantages in terms of speed, ease of integration into standard workflows, and relatively high purity. However, their reliance on non‐exclusive, membrane‐bound markers—such as tetraspanins (CD9, CD63, and CD81) and multivesicular body‐associated proteins like syntenin‐1—limits their capacity to resolve subtype‐specific vesicles. As no individual marker is exclusively associated with a single biogenetic pathway, the precise separation of exosomes from ectosomes remains elusive. Accordingly, the ISEV recommends using the general term “EVs” instead of subtype‐specific terms like “exosomes” or “ectosomes”.^[^ ^29^ ^]^ This is exemplified by the fact that what researchers have historically labeled as “exosomes” in many studies actually represents a diverse collection of small EVs that may include both endosome‐derived vesicles and small ectosomes budded directly from the plasma membrane.

Despite challenges in classification, considerable attention has been consistently directed toward elucidating functional differences among EV populations.^[^ ^58^ ^]^ A key 2017 study systematically compared the immunostimulatory properties of DC‐derived small EVs (commonly referred to as exosomes) and larger EVs (commonly called microvesicles), both loaded with ovalbumin (OVA).^[^ ^59^ ^]^ The smaller EVs demonstrated superior capacity to induce antigen‐specific IgG responses and interferon‐gamma (IFN‐γ) production. Furthermore, only mice treated with the smaller EVs showed significant expansion of OVA‐specific CD8^+^ T cells. This enhanced immunogenicity was attributed to the selective enrichment of antigens within these vesicles, a feature associated with their origin in antigen‐processing compartments.^[^ ^60^ ^]^ Their endosomal derivation from MVBs contributes to both the high antigen content and the superior immunostimulatory activity observed in the smaller EV fraction.

According to the ExoCarta database, EVs encompass a vast repertoire of over 41 000 proteins, 7 500 RNA species, and 1 100 lipid molecules, though specific cargo varies significantly based on cellular origin and physiological context.^[^ ^61^ ^]^ Importantly, EV secretion can occur either constitutively or in response to environmental stimuli, which significantly impacts the loaded condition of cargos into EVs.^[^ ^32^, ^62^ ^]^ This emphasizes the need to carefully select both the extrinsic factors and the harvest timing, as the biogenetic pathway dictates the molecular cargo composition and, consequently, the resulting immunological outcomes.^[^ ^63^ ^]^

Integrating knowledge of EV biogenesis into engineering considerations is essential, as the molecular composition of EVs—shaped not only by their cellular origin but also by the specific biogenetic pathways and physiological conditions under which they are formed—critically determines the efficiency of antigen loading, particularly in the context of endogenous engineering strategies. Collectively, these insights demonstrate that EVs from the same cellular source may exhibit substantial functional differences based on isolation parameters and physical characteristics. Therefore, rational selection of EV isolation methods, combined with optimized antigen‐loading conditions or strategies and thorough characterization, is critical for advancing EV‐based immunotherapies.

### 2.2 Approaches for Engineering Antigen‐Enriched EVs

**Figure 1:**
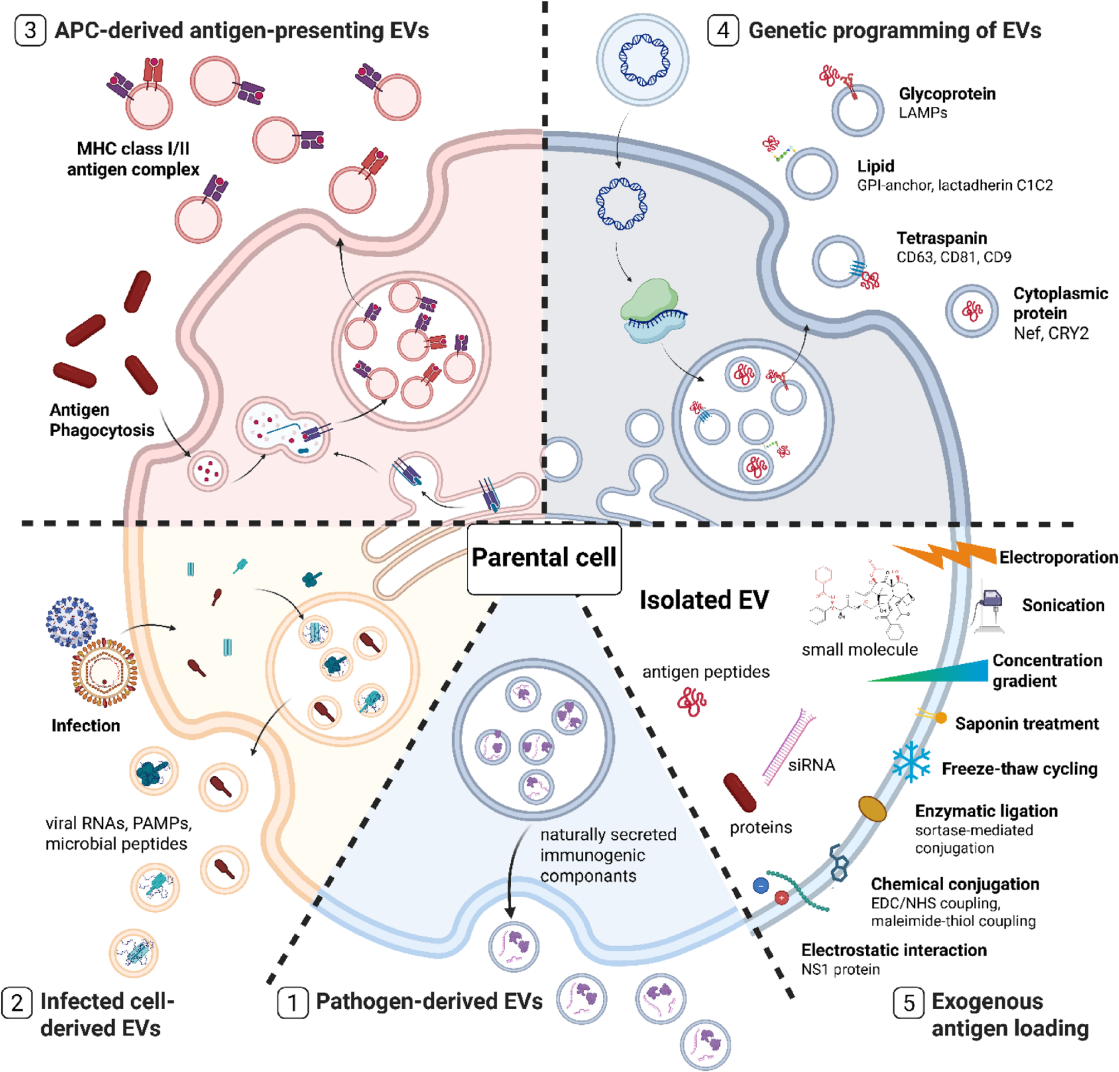
Schematic overview of diverse strategies for antigen loading into EVs 1) Pathogen‐derived EVs intrinsically encapsulate pathogen‐associated antigens and are naturally secreted from pathogens such as fungi, parasite or bacteria. 2) Infected cell‐derived EVs released from host cells upon pathogenic infection carry endogenously processed antigens with pathogen‐specific molecular signatures. 3) Antigen‐presenting cell (APC)‐derived EVs originate from professional immune cells internalizing phagocytosed exogenous antigens and uniquely present MHC‐peptide complexes on the surface of secreted vesicles. 4) Genetically engineered EVs are generated by fusing target antigens to their membrane proteins, directing them to lipid raft microdomains, or expressing cytosolic proteins with EV‐sorting motifs, enable their preferential incorporation during EV biogenesis. 5) Exogenously loaded EVs are produced by applying physical or chemical techniques—such as electroporation, sonication, or covalent conjugation—to pre‐isolated EVs, enabling antigen incorporation.

A central challenge in EV‐based vaccine development is efficiently incorporating antigens in a manner that is immunologically meaningful. Broadly, two principal antigen‐loading strategies have been established: endogenous loading, where antigens are incorporated into EVs through the donor cell's biosynthetic machinery before secretion, and exogenous loading, where antigens are introduced into or onto EVs after isolation using physical, chemical, or enzymatic methods. These approaches allow for tailored control of antigen localization, structure, and immunogenicity, thereby expanding the versatility of EVs as innovative vaccine platforms (**Figure** **1**).

#### 2.2.1 Endogenous Antigen Loading

Endogenous loading leverages the cell's intrinsic antigen processing, membrane trafficking, and EV biogenesis machinery to incorporate immunologically relevant cargo into EVs.^[^ ^64^ ^]^ This approach enables antigens to be packaged and presented in their native conformational states, within a physiological context that closely mimics natural infection or antigen exposure. Through the exploitation of MVB formation and membrane‐sorting pathways, antigens can be loaded either into the intraluminal space or onto the surface of EVs. This process has gained attention as a strategy for generating potent vaccines that capitalize on the immune‐communicative properties of EVs.^[^ ^65^ ^]^ Four representative mechanisms have been characterized to date:

##### Pathogen‐Derived EVs

Many eukaryotic pathogens—including fungi,^[^ ^66^ ^]^ protozoa,^[^ ^67^ ^]^ and helminths^[^ ^68^ ^]^—naturally secrete EVs throughout stages of growth and host infection. Pathogen‐derived EVs (P‐EVs) are released constitutively in response to diverse physiological and pathological stimuli such as immune activation, cellular stress, and infection.^[^ ^69^ ^]^ These P‐EVs are often enriched with a diverse repertoire of immunogenic components, including surface glycoproteins, nucleic acids, pigments, and lipids, which reflect the physiological state and antigenic complexity of the parent organism. Their polyantigenic content enables the induction of broad immune responses that are potentially more resistant to immune escape mechanisms. For example, Rizzo et al. reported that EVs derived from *Cryptococcus neoformans* induced strong IgG responses and significantly prolonged survival in infected mice without the use of adjuvants, demonstrating the immunogenic potential of fungal EVs for vaccine development.^[^ ^70^ ^]^ These P‐EVs may function as in situ vaccines by engaging innate immune receptors and efficiently delivering diverse pathogen‐associated antigens to APCs for robust immune priming.

Importantly, this combinatorial antigen presentation can elicit broad immune responses less susceptible to antigenic variation or immune escape, common limitations of conventional single‐target therapeutics. In contrast to such agents, pathogens or tumor cells have difficulty developing resistance to the diverse antigenic composition of EVs, making EV‐based strategies highly attractive for developing durable, mutation‐resilient vaccines.

##### Infected Cell‐Derived EVs

During microbial and viral infections, host cells commonly produce EVs enriched with pathogen‐derived components, including viral RNAs, pathogen‐associated molecular patterns (PAMPs), and microbial peptides.^[^ ^71^ ^]^ These EVs are generated via endogenous sorting mechanisms, trafficking intracellular pathogen components into MVBs for release as EVs. For instance, EVs isolated from SARS‐CoV‐2‐infected lung epithelial cells were shown to carry viral RNA and spike protein fragments, and were capable of activating DCs and inducing antiviral T cell responses in vitro and in vivo.^[^ ^72^ ^]^

Infection‐derived EVs reflect the dynamic intracellular state of infected cells, functioning both as antigen carriers and immunological messengers.^[^ ^73^, ^74^ ^]^ This phenomenon can be viewed from two contrasting perspectives. While host cells utilize infection‐derived EVs to disseminate antigenic signals and activate antiviral immunity, many viruses have evolved mechanisms to hijack the exosomal pathway, cloaking their antigens, subverting immune detection, and facilitating intercellular spread.^[^ ^75^ ^]^ These contrasting immune‐modulatory properties have inspired efforts to therapeutically harness or mimic infection‐associated EV dynamics. Specifically, tumor cells infected with oncolytic viruses or transduced with viral vectors produce EVs that carry tumor antigens along with endogenous danger‐associated signals or engineered immune‐stimulatory cues, thereby significantly enhancing immunogenicity.^[^ ^76^ ^]^ These EVs activate innate immune receptors such as TLR3 and RIG‐I, concurrently delivering TAAs to APCs for efficient cross‐priming.

In both infectious and oncogenic contexts, EV secretion is enhanced under stress conditions—including viral replication, DNA damage, hypoxia, and inflammatory cytokine exposure—enabling context‐specific EV cargo loading.^[^ ^77^ ^]^ This context‐adaptive behavior of infection‐derived EVs offers a significant advantage over conventional subunit vaccines, which often require external adjuvants and lack spatial or temporal cues relevant to in vivo infection.^[^ ^78^ ^]^ In contrast, EVs derived from infected or transduced cells inherently co‐deliver antigenic and inflammatory signals, potentially reducing the need for synthetic adjuvants and better recapitulating the immunological context of natural pathogen or tumor recognition.

##### APC‐Derived Antigen‐Presenting EVs

APCs, such as DCs and macrophages, can endogenously load antigens into EVs following the phagocytic uptake of exogenous materials—a process referred to as indirect antigen pulsing. Upon antigen internalization, APCs process foreign proteins via the endosomal‐lysosomal pathway and present peptide fragments on MHC class I or II molecules.^[^ ^79^ ^]^ During this process, MHC‐peptide complexes, along with co‐stimulatory molecules such as CD86 and ICAM‐1, are incorporated into intraluminal vesicles and subsequently secreted as immunologically active EVs.^[^ ^80^ ^]^ EV release from APCs could be further enhanced by immunogenic stimuli such as TLR activation, IFN‐γ exposure, or cytokine‐induced maturation, allowing the antigen‐loaded vesicles to reflect the immune status of the parental cell.^[^ ^43^ ^]^

EVs derived from APCs offer the advantage of presenting antigens within a membrane‐embedded, immunologically active context that supports direct T cell recognition without requiring further antigen processing by host APCs. For instance, EVs released by murine DCs pulsed with OVA peptides have been shown to activate antigen‐specific CD8^+^ and CD4^+^ T cells, demonstrating their ability to mediate antigen presentation and prime adaptive immunity.^[^ ^81^ ^]^ In addition to antigen display, these vesicles are enriched with co‐stimulatory ligands, immunomodulatory molecules, and adhesion factors—such as CD86, ICAM‐1, and MHC complexes—that further enhance their capacity to prime robust immune responses and optimize their trafficking to lymphoid tissues and engagement with immune cells.^[^ ^65^, ^82^, ^83^, ^84^, ^85^ ^]^

This intrinsic ability of APC‐derived EVs to carry preloaded MHC‐peptide complexes has been translated into cancer vaccine design. In the phase II trial reported by Besse et al., clinical‐grade DC‐derived exosomes (DEXs) were produced from monocyte‐derived DCs (Mo‐DCs) that were matured with IFN‐γ and loaded with MHC class I‐ and class II‐restricted tumor‐associated peptides (including MAGE‐A1, MAGE‐A3, NY‐ESO‐1, and MART‐1), prior to EV harvesting, thereby utilizing an endogenous antigen‐loading strategy through the natural antigen processing pathways of DCs.^[^ ^86^ ^]^ Although the treatment demonstrated an acceptable safety profile and modest clinical benefits, the study did not meet its primary endpoint, and tumor‐specific T cell responses were not detected, indicating the need for further optimization of immunogenicity and therapeutic efficacy. Possible contributing factors include suboptimal antigen selection, IFN‐γ‐induced PD‐L1 expression during DEX manufacturing, and the need for combination with checkpoint blockade to overcome tumor immunosuppression.

Nevertheless, the inherent combination of antigenic and immunostimulatory signals makes APC‐derived EVs particularly potent and promising for eliciting both primary and memory immune responses, especially in immunocompromised individuals with impaired APC functionality.

##### Genetic Programming of EVs

Genetic engineering enables the loading of antigens into EVs by modifying producer cells at the DNA level, allowing not only the incorporation of specific antigens, but also the modulation of their expression levels and trafficking pathways. Among these strategies, a widely adopted strategy involves transfecting producer cells with DNA constructs encoding either antigens alone or antigens fused to membrane‐targeting or scaffolding motifs. A representative example involves fusing antigens to full transmembrane domains which facilitates their stable insertion into the lipid bilayer and subsequent external presentation on the vesicle surface commonly referred to as the transmembrane‐fused antigen approach. In particular, membrane proteins that are abundantly enriched in EVs, such as lysosome‐associated membrane protein 2 (LAMP‐2) or tetraspanin (CD63) are effectively directed into the EV biogenesis pathway, thereby serving as domains that guide the fused antigens through MVBs and into secreted EVs. As an example, fusion of HER2 with LAMP‐2, which promotes endosomal/lysosomal trafficking and efficient incorporation into EV surface, ultimately enabling their targeted uptake by recipient cells via epidermal growth factor receptor (EGFR)‐mediated endocytosis.^[^ ^87^ ^]^

The transmembrane‐fused antigen approach has been used to deliver antigens not only on the external surface of EVs but also on their internal membrane, thereby enabling more efficient intravesicular loading of fusion antigens. For instance, the Nef^mut^‐E7 fusion antigen platform combines a mutated, nonpathogenic form of the HIV Nef protein with the human papillomaviruses E7 antigen, promoting efficient incorporation of E7 into EVs produced by muscle cells in vivo.^[^ ^88^ ^]^ Notably, the Nef^mut^ scaffold facilitates intravesicular encapsulation of the fused antigen by exploiting endogenous EV biogenesis pathways. This platform has been shown to elicit potent E7‐specific CD8^+^ T cell responses in mice, illustrating the translational potential of genetically engineered EV‐based vaccines. In parallel, the efficiency of antigen loading can similarly be modulated by selecting optimal sorting motifs—such as LAMP‐1,^[^ ^89^ ^]^ CD63,^[^ ^90^ ^]^ or WW domain–PPxY motif interactions^[^ ^91^ ^]^—which direct cargo through distinct intracellular pathways and influence accessibility to MHC class I or II presentation. Moreover, transfected cells can be engineered to coexpress immunostimulatory molecules, such as IL‐12^[^ ^92^, ^93^ ^]^ or GM‐CSF,^[^ ^94^, ^95^ ^]^ enhancing the overall adjuvanticity of the EV platform.

Lipid anchoring strategies offer a complementary route of enriching antigens on the EV membrane, whereby antigens are genetically fused to lipid‐associating motifs to facilitate stable surface localization independent of conventional endosomal trafficking.^[^ ^96^, ^97^, ^98^ ^]^ These strategies emulate biologically efficient modes of protein‐membrane association inspired by naturally occurring lipidation processes, wherein lipidation signals facilitate the insertion of a single lipid moiety into the inner leaflet of the EV membrane and anchor functional proteins.^[^ ^99^ ^]^ One well‐established method employs glycosylphosphatidylinositol (GPI) signal sequences; anti‐EGFR nanobodies fused to GPI‐anchor motifs have been successfully presented on EV surfaces while preserving binding specificity and enhancing vesicle targeting to EGFR‐positive tumor cells. A complementary strategy leverages the phosphatidylserine‐binding C1C2 peripheral domain of lactadherin.^[^ ^100^, ^101^, ^102^ ^]^ Rountree et al. employed this approach to anchor TAAs, such as PSA and PAP, to EVs, inducing strong antigen‐specific T cell responses and therapeutic tumor control in murine models.^[^ ^100^ ^]^ This platform has since been extended to antigens such as HER2 and Carcinoembryonic antigen (CEA),^[^ ^101^ ^]^ demonstrating enhanced immunogenicity and tumor suppression even in antigen‐tolerant transgenic models.

In addition to strategies that directly couple antigens to membrane‐targeting or scaffolding motifs, alternative approaches have also been employed to enhance vesicular antigen output by modulating the fundamental biogenesis and intracellular trafficking machinery of the producer cells. One such strategy involves the overexpression of Rab27a, a small GTPase critically involved in exosome secretion.^[^ ^103^ ^]^ Engineering tumor cells to stably overexpress Rab27a significantly increases the yield of exosomes enriched in canonical markers such as CD63 and Hsp70, enhancing their immunostimulatory potential. Notably, exosomes derived from Rab27a‐overexpressing lung cancer cells not only promote upregulation of MHC class II, CD80, and CD86 on DCs, but also facilitate CD4^+^ T cell proliferation and type I cytokine secretion, culminating in effective tumor growth inhibition in vivo. Similarly, genetic introduction of the class II transactivator (CIITA), a master regulator of MHC class II gene expression, enables tumor cells to produce EVs enriched in MHC class II and TAAs.^[^ ^104^, ^105^ ^]^ CIITA‐transduced melanoma cells secrete vesicles (CIITA‐Exo) that are enriched in both MHC class II and TAAs such as TRP‐2, and these vesicles have been shown to activate DCs and stimulate both CD4^+^ and CD8^+^ T cell responses in vivo, resulting in significantly enhanced tumor rejection.^[^ ^104^ ^]^ These upstream engineering strategies do not alter the antigen structure per se but strategically enhance the quantity or immunological breadth of antigenic material processed into EVs, offering a synergistic layer to conventional loading strategies.

Together, these endogenous engineering strategies—whether through intracellular sorting or surface anchoring—underscore the versatility of genetic programming approaches for functionalizing EVs. By enabling topologically accurate, membrane‐embedded antigen presentation and co‐display of adjuvant or targeting ligands, genetic engineering supports robust T and B cell engagement. Extending from these findings, genetic interventions can be further optimized or strategically combined—through enhanced vesicle secretion or expanded antigen presentation pathways—to fine‐tune the immunogenic potential of EVs. Nonetheless, achieving precise control over antigen density, spatial orientation, and presentation stability remains a critical challenge for maximizing therapeutic efficacy.

#### 2.2.2 Exogenous Antigen Loading

Exogenous antigen loading offers an alternative strategy for functionalizing EVs as vaccine carriers, in which antigens are incorporated into or onto EVs following their isolation from donor cells. This post‐production modification circumvents the need for genetic manipulation of producer cells and enables the flexible loading of diverse antigenic cargo, including proteins, peptides, nucleic acids, and even small molecules. Such modularity enables optimization of antigen composition, concentration, and offers the potential to preserve structural integrity, although this remains a technical challenge requiring meticulous methodological optimization.

A range of physical, chemical, and biological methods has been developed to facilitate exogenous loading.^[^ ^106^, ^107^ ^]^ Among these, electroporation is the most widely employed technique. By applying controlled electrical pulses, transient pores form in the EV membrane, permitting the direct entry of biomolecules, particularly charged biomolecules (e.g., mRNA or siRNA), which are especially amenable to this method.^[^ ^87^, ^106^, ^108^, ^109^, ^110^, ^111^, ^112^ ^]^ This method has proven highly effective in loading EVs with antigen‐encoding mRNA, offering a hybrid strategy that combines the benefits of genetic immunization with EV‐mediated delivery.^[^ ^106^, ^108^ ^]^ In addition to electroporation, a variety of complementary techniques have been explored to facilitate passive diffusion or mechanical incorporation of antigens into EVs. These include sonication,^[^ ^113^, ^114^ ^]^ freeze‐thaw cycling,^[^ ^115^ ^]^ extrusion,^[^ ^116^, ^117^ ^]^ and saponin‐mediated membrane permeabilization,^[^ ^118^ ^]^ and membrane fusion strategies involving LNPs or other cell‐derived membranes.^[^ ^119^, ^120^, ^121^ ^]^ For instance, repeated freeze‐thaw cycling destabilize the vesicle membrane, allowing encapsulation of protein or peptide antigens within the EV lumen.^[^ ^115^ ^]^ Likewise, extrusion physically incorporates antigens by forcing EVs and cargo through defined pore‐size membranes, while maintaining vesicle morphology. In addition, PEG‐mediated incubation facilitates direct membrane fusion between EVs and LNPs or cell‐derived membranes, enabling efficient cargo transfer.^[^ ^122^ ^]^ Notably, this approach has also been shown to enhance circulatory stability, potentially by promoting tighter membrane‐protein packing and reducing opsonization. More recently, DNA‐guided membrane fusion has emerged as a programmable strategy for achieving targeted bilayer merging and cargo delivery.^[^ ^123^ ^]^ Together, these physical and fusion‐based techniques broaden the spectrum of applicable techniques for exogenous antigen loading, offering adaptable and efficient approaches that preserve EV structural integrity while maximizing antigen encapsulation.

Surface modification of EVs offers an additional dimension of engineering flexibility. The lipid bilayer and associated membrane proteins offer structured anchoring platforms for antigen display through mechanisms such as lipid insertion (e.g., GPI anchor, DSPE‐PEG),^[^ ^124^, ^125^, ^126^ ^]^ whereby antigens are incorporated into the membrane by embedding lipid moieties; affinity‐based interactions (e.g., Streptavidin–biotin,^[^ ^102^ ^]^ CP05‐CD63^[^ ^127^, ^128^ ^]^), relying on noncovalent binding between antigens and membrane‐resident ligands; chemical conjugation^[^ ^129^, ^130^ ^]^ (e.g., EDC/NHS coupling, maleimide‐thiol coupling), which covalently links antigens to membrane components through reactive functional groups; enzymatic ligation (e.g., Sortase A‐mediated ligation^[^ ^131^ ^]^), utilizing enzymes like transpeptidases to catalyze site‐specific attachment of antigens to vesicular surfaces; and metabolic labeling^[^ ^132^, ^133^ ^]^ (e.g., Azide–alkyne click chemistry), in which modified biosynthetic precursors are incorporated into membrane structures to enable subsequent conjugation of antigens via bioorthogonal chemistry.

Importantly, these surface‐engineering strategies are applicable to a broad range of antigen formats—from full‐length proteins to short peptides. In particular, advanced chemical techniques such as bioorthogonal click chemistry and enzymatic ligation have demonstrated enhanced immunogenicity and targeting capacity by enabling site‐specific and stable conjugation of immunologically active or targeting moieties onto the EV membrane. A notable example was reported by Zhu et al., who engineered DEX with surface‐conjugated dibenzylcyclooctyne (DBCO) moieties through chemical crosslinking.^[^ ^130^ ^]^ This modification facilitated a copper‐free click reaction between DBCO‐functionalized DEX (DEX‐DBCO) and the azide‐tagged glycopeptide antigen MUC1‐N_3_, resulting in stable surface presentation of the antigen. The resulting MUC1‐decorated DEX elicited the production of MUC1‐specific IgG antibodies exhibiting high‐affinity binding toward MUC1‐overexpressing tumor cells, thereby promoting potent antigen‐specific immune responses in vivo.

One representative example illustrating the potential of surface‐engineered EVs involves the use of tumor‐specific peptide neoantigens conjugated to CP05, a synthetic anchor peptide with high affinity for CD63^[^ ^127^ ^]^ Through co‐incubation, CP05‐fused neoantigens were selectively and stably anchored onto the EV membrane. Notably, this strategy allowed for the flexible incorporation of multiple MHC class I‐ or II‐restricted peptide neoantigens (M21, M27, and M33), which facilitated efficient DC‐mediated antigen presentation, thereby enhancing the magnitude of CTL responses and promoting tumor regression. When combined with anti‐PD‐1 checkpoint blockade, this approach further achieved complete tumor eradication and established durable immunological memory. These findings highlight a modular and adaptable design framework applicable to diverse tumor antigens across various cancer types, thereby broadening the immunogenic potential of the platform.

Moreover, early‐phase studies have demonstrated the clinical feasibility of this approach. In the phase I trial by Morse et al., synthetic MAGE peptides (MAGE‐A3, ‐A4, ‐A10, and MAGE‐3DPO4) were directly conjugated to purified EVs following their isolation from Mo‐DC cultures, thereby enabling controlled antigen display via the exogenous MHC class I pathway.^[^ ^134^ ^]^ A comparable strategy was employed by Escudier et al.,^[^ ^135^ ^]^ in which GMP‐grade EVs were post‐loaded with a class I‐restricted MAGE‐A3 peptide under mild acidic conditions performing higher number of peptide‐MHC complex.^[^ ^136^ ^]^ These post‐isolation methods, collectively termed direct antigen pulsing, allow precise antigen display—typically by externally coupling class I‐restricted peptides. In both studies, these exogenous approaches were evaluated alongside conventional endogenous loading methods to enable direct immunological comparison. Preclinical analyses between the two methods demonstrated that EVs directly loaded with synthetic peptides elicited superior immunogenicity compared to those derived from peptide‐pulsed DCs, underscoring the prominent immunogenicity and translational promise of direct exogenous peptide loading.

Recent advances in microfluidic engineering have enabled the development of innovative platforms for exogenous antigen loading onto EVs, offering enhanced efficiency, scalability, and precision over traditional ultracentrifugation‐based methods. Notably, Krivitsky et al. introduced an electrochemically controlled microfluidic device that integrates immunoaffinity capture, nucleic acid loading, and controlled release of EVs in a single streamlined workflow.^[^ ^137^ ^]^ EVs are selectively immobilized on antibody‐coated micro‐carbon fiber electrodes and subsequently released by applying a negative potential, which simultaneously induces electrostatic repulsion, generates hydrogen and oxygen gases through electrolysis, and alters local pH—three effects that collectively facilitate to gently detach the EVs. During immobilization, cationic polyplexes (e.g., poly(ethyleneimine)–siRNA) can be flowed through the microchannel, allowing for simultaneous EV purification—by retaining only antibody‐bound EVs while washing away unbound contaminants—and cargo loading in a single step. Compared to conventional methods, this “all‐in‐one” device significantly reduces reagent waste, minimizes contaminant carryover, and improves consistency of engineered EV products. Complementing this, Zhao et al. developed a polydimethylsiloxane (PDMS)‐based microfluidic cell culture platform that enables continuous EV collection, surface modification, and light‐triggered release of intact MHC‐I antigen‐decorated EVs.^[^ ^138^ ^]^ By culturing leukocytes on‐chip, EVs are continuously secreted into the microfluidic channel, where tumor antigen peptides—linked to magnetic beads via a photo‐cleavable linker—are anchored in place. These magnetic beads capture the MHC‐I–decorated EVs in real time. Upon UV exposure, the linkers are cleaved, releasing intact EVs while preserving the MHC‐peptide complexes on their surface.

In summary, exogenous antigen loading offers a uniquely modular and adaptable strategy for engineering EV‐based vaccines, enabling dynamic customization tailored to clinical requirements. Continued advances in loading methodologies, along with the establishment of standardized and scalable production frameworks, will be critical for unlocking the full therapeutic potential of exogenously engineered EVs across cancer, infectious diseases, and broader immunological applications.

### 2.3 Characterization Tools for EV‐Based Vaccine Development

A wide array of analytical technologies has been established to precisely validate key parameters of EVs—including structural integrity, surface antigenicity, and cargo composition—which are essential not only for ensuring batch‐to‐batch consistency and therapeutic efficacy but also for advancing their clinical translation as vaccine platforms.

EV characterization strategies, as defined in the 2023 minimal information for studies of extracellular vesicles (MISEV) guidelines,^[^ ^54^ ^]^ are broadly categorized into three methodological domains: 1) physicochemical analysis, which assesses the structural and biophysical properties of EVs and includes nanoparticle tracking analysis (NTA),^[^ ^139^ ^]^ dynamic light scattering (DLS),^[^ ^140^ ^]^ tunable resistive pulse sensing (TRPS),^[^ ^141^ ^]^ and asymmetric flow field‐flow fractionation (AF4)^[^ ^142^ ^]^ which offer quantitative profiling of size distribution and concentration, thereby facilitating quality control across heterogeneous EV populations; electrophoretic light scattering (ELS)^[^ ^143^ ^]^ for surface charge and zeta potential determination; as well as transmission electron microscopy (TEM),^[^ ^144^ ^]^ cryo‐electron microscopy (cryo‐EM),^[^ ^145^ ^]^ scanning electron microscopy (SEM),^[^ ^146^ ^]^ and atomic force microscopy (AFM)^[^ ^147^ ^]^ for assessing morphology and verifying vesicle integrity; 2) molecular profiling, which identifies intra‐ and extravesicular components such as proteins, lipids, and nucleic acids using tools such as Western blotting,^[^ ^148^ ^]^ flow cytometry (FCM),^[^ ^149^ ^]^ and fluorescence‐based assays (FBA);^[^ ^150^ ^]^ and 3) functional assays, which evaluates the biological competence and immunological impact of EV‐encapsulated cargos.^[^ ^151^ ^]^

Among these, several classical techniques are widely adopted across EV studies. For example, TEM, cryo‐EM, and SEM are routinely employed for high‐resolution imaging to verify vesicle integrity and morphology, while NTA, AF4, and TRPS serve as robust tools for quantifying particle size and concentration across heterogeneous EV populations. Additionally, protein‐level characterization is commonly performed using Western blotting, whereas immunophenotyping approaches such as conventional and imaging‐based FCM enable multiplexed profiling of EV surface markers through fluorescent labeling. These widely used methods continue to serve as foundational tools for EV research, offering both accessibility and reliability in capturing core structural and molecular characteristics.

To enable more precise and mechanistic interrogation of EV‐based vaccine platforms, advanced analytical technologies have emerged that offer nanoscale and functional resolution, facilitating the accurate quantification of both surface and intraluminal cargos and markers even at low abundance.^[^ ^152^, ^153^ ^]^ AFM allows for topographic mapping and receptor localization at the single‐vesicle level, particularly useful for analyzing nanoscale surface architecture and biophysical properties of EVs. Beyond receptor profiling, AFM‐based nanoindentation has revealed that typical EVs range from 40 to 160 nm in diameter and exhibit stiffness values across a broad range of Young's modulus values (≈0.1–25 MPa).^[^ ^154^ ^]^ These physical parameters—including size, stiffness, morphology, and surface charge—have been shown to influence cellular uptake efficiency, underscoring their potential as predictive indicators of delivery performance.^[^ ^155^ ^]^ Extending these analyses into molecular interactions, label‐free biosensing techniques such as surface plasmon resonance (SPR)^[^ ^156^ ^]^ and surface‐enhanced Raman spectroscopy (SERS)^[^ ^157^ ^]^ permit dynamic tracking of antigen‐receptor interactions and binding kinetics. Likewise, advanced fluorescence‐based methods—including fluorescence correlation spectroscopy (FCS),^[^ ^158^ ^]^ total internal reflection fluorescence microscopy (TIRFM), and single EV analysis^[^ ^159^ ^]^—offer highly sensitive subpopulation quantification. Digital detection platforms such as single‐particle interferometric reflectance imaging sensors (SP‐IRIS) (SP‐IRIS; e.g., ExoView) and electrochemical sensing systems provide ultrasensitive readouts of both surface‐bound and intraluminal cargos, often using minimal sample volumes.^[^ ^160^, ^161^ ^]^

Although many EV studies have traditionally relied on a single surface marker such as CD63, recent findings suggest that this approach is increasingly considered insufficient for evaluating vesicle purity and compositional consistency.^[^ ^29^ ^]^ Accordingly, multiparametric biophysical indicators—such as protein‐to‐lipid ratio and membrane fluidity^[^ ^162^ ^]^—have gained increasing attention, along with emerging single‐vesicle analytical technologies such as interferometric NTA (iNTA), high‐resolution FCM, and AFM‐based nanoindentation, which together enable more rigorous and quantitative assessment of EV heterogeneity, structural integrity, and batch reproducibility. Taken together, these techniques form a hierarchical framework—from fundamental physicochemical assays to advanced single‐vesicle analytics—that enables comprehensive validation of EV cargo integrity, immunogenicity, and delivery potential. As the field moves toward clinical application, the integration of these complementary approaches will be essential for ensuring product consistency, mechanistic understanding, and translational readiness.

### 2.4 From Systemic Delivery to Cellular Uptake of EV‐Based Vaccines

The successful implementation of EV‐based vaccines hinges not only on the efficient loading of immunogenic molecules but also on their precise uptake by target immune cells. Administration route critically determines the biodistribution, uptake mechanism, and immune outcome of EV formulations.

Oral delivery of EV vaccines represents a practical and needle‐free strategy, particularly when leveraging the intrinsic stability of plant‐derived EVs.^[^ ^163^, ^164^, ^165^, ^166^ ^]^ Extracted from edible sources such as orange, these nanovesicles exhibit natural enrichment in bioactive lipids, RNAs, and proteins, while demonstrating exceptional resistance to enzymatic degradation and gastric pH. In a representative study, orange‐derived EVs (oEVs) encapsulating SARS‐CoV‐2 mRNA protected the cargo through the digestive tract, enabled transintestinal uptake, and successfully elicited both systemic IgG and mucosal IgA responses in mice.^[^ ^164^ ^]^ The EV‐cell interaction in this context primarily involved uptake by intestinal epithelial cells and gut‐associated lymphoid tissue, leading to downstream activation of splenic CD4^+^ and CD8^+^ T cells. Notably, the stability of lyophilized oEVs for over 12 months at room temperature further underscores their practical potential for oral vaccine deployment. Their stability at ambient temperatures and compatibility with oral and intranasal administration make them attractive candidates for needle‐free, cold‐chain‐independent vaccination—critical features for global immunization efforts. Beyond plant‐derived sources, milk‐derived EVs have also demonstrated notable resilience under gastric conditions, further broadening the scope of EV‐based oral delivery platforms aimed at inducing both localized and systemic immune responses.^[^ ^167^ ^]^ Additionally, red blood cell‐derived EVs, originating from anucleate cells, offer advantages by eliminating concerns of nuclear DNA contamination, suggesting further potential for safe and scalable vaccine delivery applications.^[^ ^168^ ^]^

Inhalational delivery targets the respiratory mucosa, a primary entry point for airborne pathogens.^[^ ^169^ ^]^ EVs derived from human lung spheroid cells, when surface‐modified with recombinant SARS‐CoV‐2 RBD, form VLPs (RBD‐Exo VLPs) that can be nebulized and delivered into the lungs. These vesicles demonstrated superior retention in lung parenchyma and enhanced uptake by airway‐resident DCs compared to liposomal formulations. Upon cellular internalization, RBD‐exo induced strong local mucosal immunity (SIgA) and Th1‐biased systemic responses involving IFN‐γ–producing CD4^+^ and CD8^+^ T cells in pulmonary compartments. These results collectively highlight the potential to facilitate transintestinal uptake and translation of encapsulated cargo, serving as components of edible or mucosally delivered vaccine platforms capable of inducing both localized and systemic immune responses.

Injectable routes, including intravenous (IV), intramuscular (IM), subcutaneous (SC), and intraperitoneal (IP) administration, offer direct systemic access and remain the most established modes for therapeutic vaccine delivery.^[^ ^170^ ^]^ In the context of EV‐based vaccines, these routes similarly enable systemic distribution, with route‐dependent differences in organ targeting and biodistribution. EVs inherently possess a homing capacity that mediates organ‐specific accumulation, not only in immune‐relevant tissues such as the spleen and lymph nodes, but also in nonimmune organs—including the liver, lung, and pancreas— depending on their cellular origin and surface molecular signatures. Among them, EVs derived from APCs have demonstrated preferential homing toward secondary lymphoid organs, including the spleen and liver. In preclinical models of hepatitis B, such APC‐derived EVs induced CD8^+^ T cell expansion and reprogrammed M2 macrophages in the hepatic microenvironment, thereby overcoming local immune suppression.^[^ ^170^ ^]^ However, despite these organ‐targeting tendencies, especially toward lymphoid tissues, EVs still exhibit uncontrolled accumulation in clearance organs such as the liver and spleen, which limits delivery efficiency. Among injectable routes, IV administration allows for rapid and widespread EV dissemination, with early pulmonary accumulation followed by prolonged hepatic retention and subsequent uptake by immune‐relevant organs such as the spleen.^[^ ^171^ ^]^ In contrast, IP injection leads to predominant accumulation in the liver, gastrointestinal (GI) tract, and pancreas within the first 24 h, with hepatic localization persisting up to 22 days post‐injection.^[^ ^172^, ^173^, ^174^ ^]^ SC and IM routes have similarly been associated with preferential delivery to the liver and GI tract. Nevertheless, certain engineered EV formulations administered via IM injection have successfully reached draining lymph nodes and the spleen, highlighting how vesicle design can critically shape immunologically relevant biodistribution profiles.^[^ ^175^ ^]^

To improve pharmacokinetics and minimize nonspecific clearance, surface engineering strategies have been introduced to extend the circulatory half‐life of EVs. A notable example is the post‐insertion of lipopolyoxazolines (LipoPOx) into EV membranes, as demonstrated by Simon et al., which offered a compelling alternative to conventional PEGylation strategy.^[^ ^176^ ^]^ This POxylation method resulted in a sixfold increase in blood half‐life at 6 h post‐injection while preserving key biological functions such as immunomodulatory capacity and tumor‐penetrating ability. Notably, LipoPOx‐modified EVs exhibited reduced hepatic and splenic uptake and enhanced tumor accumulation compared to PEGylated EVs, suggesting that rational surface modification can significantly improve therapeutic EV performance.

Furthermore, targeted engineering approaches are increasingly essential to overcome biodistribution barriers and enhance antigen delivery to immunologically relevant or disease‐relevant sites. Advances in EV bioengineering have enabled the functionalization of vesicle membranes with ligands—such as antibodies, nanobodies, peptides, and aptamers—that drive receptor‐specific uptake by immune or tumor‐associated cells. For instance, Wiklander et al. developed EVs displaying Fc‐binding domains on their membranes, which allowed modular attachment of therapeutic antibodies such as trastuzumab or atezolizumab.^[^ ^177^ ^]^ These antibody‐decorated EVs demonstrated enhanced receptor‐specific uptake in HER2^+^ breast cancer and PD‐L1^+^ melanoma cells, achieving up to a 509‐fold increase in cellular internalization and improved in vivo tumor accumulation. Similarly, Pham et al. utilized enzyme‐mediated conjugation strategy to covalently attach EGFR‐targeting peptides and nanobodies onto EV surfaces, enabling selective binding to EGFR‐overexpressing tumors.^[^ ^131^ ^]^ Additional strategies include genetic fusion of tumor‐penetrating peptides such as iRGD to the exosomal membrane protein Lamp2b, as reported by Tian et al.^[^ ^178^ ^]^ These iRGD‐functionalized EVs selectively targeted αv integrin–expressing breast cancer cells, efficiently delivered doxorubicin payloads, and significantly suppressed tumor growth in murine models without inducing systemic toxicity. Wan et al. further introduced a nongenetic method by conjugating the nucleolin‐binding aptamer AS1411 to PEGylated cholesterol and incorporating it into EVs via mechanical extrusion.^[^ ^179^ ^]^ These aptamer‐grafted EVs enabled rapid and selective uptake into nucleolin‐positive breast cancer cells, and their aptamer‐mediated delivery of paclitaxel yielded greater antitumor efficacy than free drug or untargeted EV controls.

Beyond cancer, EV targeting strategies have also shown promise in treating nonmalignant diseases. In a mouse model of acute liver failure, Kim et al. engineered EVs to express SIRPα, a CD47‐binding ligand, enabling preferential accumulation in CD47‐overexpressing necroptotic hepatocytes.^[^ ^180^ ^]^ These SIRPα‐EVs facilitated immune clearance of damaged cells via macrophage reprogramming and promoted hepatocyte regeneration, exemplifying the dynamic capacity of engineered EVs to reshape tissue microenvironments in vivo.

Taken together, combining precise control over the route of administration with rational EV surface engineering provides a powerful approach to direct biodistribution and elicit desired immunological responses tailored to specific therapeutic goals. Whether targeting mucosal surfaces through oral or inhalational delivery, or accessing systemic immune compartments via injection, each pathway influences how EVs reach and are internalized by key immune cell populations. Further governed by EV surface composition—such as lipids, proteins, and glycoconjugates—these interactions are finely tuned by cell‐specific recognition and uptake processes that ultimately influence vaccine efficacy. Moreover, the EV's cellular origin, inherent immunogenicity, and modifiability via surface engineering or antigen loading all contribute to its functional behavior. As such, the rational design of EV‐based vaccines necessitates not only immunogenic potency but also precise delivery kinetics, minimizing systemic clearance and enhancing localization to immunologically active or disease‐relevant sites—objectives fundamentally dependent on sophisticated EV bioengineering to optimize surface features, cellular origin, and administration route for maximal immune engagement.

## 3 EV‐Based Cancer Vaccine

Cancer immunotherapy has emerged as a pivotal strategy in contemporary oncology, focusing on modulating the host immune system to reinvigorate antitumor responses. Although immune checkpoint blockade (ICB) and adoptive T‐cell therapies currently dominate clinical applications, therapeutic cancer vaccines—including those based on EV platforms—have garnered particular attention due to their potential for high specificity and durable immune memory (**Table** **1**).^[^ ^181^, ^182^, ^183^ ^]^ Unlike conventional prophylactic vaccines, which aim to prevent infections by priming the immune system against pathogens, therapeutic cancer vaccines are designed to elicit robust and tumor‐specific immune responses, ultimately promoting the eradication of malignant cells and preventing disease recurrence. Therapeutic cancer vaccines exert their effects by inducing both humoral and cellular immune responses, with a particular emphasis on activating CTLs.

**Table 1:** EV‐based cancer vaccines under preclinical and clinical investigation.

Source of EV	Disease		Antigen	Loading method	Clinical trial	Ref.
DC‐derived EVs	IFNγ‐matured DCs from NSCLC patients	NSCLC (stage IIIb/IV)b)	MAGE.A1, MAGE.A3, NY‐ESO‐1, Melan‐A/MART1, MAGE.A3.DP04, EBV pan‐DR	Antigen pulsing (indirect)a)	Phase II/ NCT01159288	[86]
	BM‐DC, Mo‐DC from melanoma patients	Metastatic melanoma (stage IIIb/IV)	MAGE3.A1, MAGE3.DP04	Antigen pulsing (direct, indirect)	Phase I	[197]
	Autologous Mo‐DC	Metastatic melanoma (stage IIIb/IV)	MAGE3.A1/B35, MAGE3.DP04	Antigen pulsing (direct, indirect)		[135]
		NSCLC (stage IIIb/IV)	MAGE.A3, MAGE.A4, MAGE.A10, MAGE3DP04	Antigen pulsing (direct, indirect)		[134]
	DC 2.4 cell	Melanoma (B16‐OVA)	OVA with anti‐CD3, anti‐EGFR antibodies	Antigen pulsing (indirect), Exogenous (DSPE anchoring)	Preclinical	[126]
		Colon cancer (MC‐38), Melanoma (B16F10)	Neoantigen peptides (M27, M30, Adpgk), OVA	Exogenous (electroporation‐mediated loading of antigen)		[112]
		Hepatocellular carcinoma (Hepa1‐6)	AFP (full‐length or peptide), OVA, GPC3	Exogenous (CP05 anchoring)		[128]
		Melanoma (B16‐MUC1)	MUC1 glycopeptide	Exogenous (chemical conjugation)		[130]
		Hepatocellular carcinoma (Hepa1‐6)	Tumor lysates (Hepa1‐6)	Tumor lysate pulsing (indirect)		[308]
		Hepatocellular carcinoma (Hepa1–6 (ectopic); Hepa1–6 (orthotopic); DEN–induced autochthonous)	AFP	Genetic engineering (AFP antigen overexpression)		[309]
	BM‐DC	Melanoma	OVA peptide with anti‐CTLA‐4 antibody	Antigen pulsing (indirect), Exogenous (DPPE anchoring)		[125]
		N/A	OVA	Antigen pulsing (direct acid‐strip, indirect)		[310]
		Melanoma (B16F10)	Tumor lysates (B16F10)	Tumor lysate pulsing (indirect)		[311]
		N/A	SIINFEKL, Gp33	Antigen pulsing (indirect)		[312]
		Glioma (GL261)	Tumor lysates (GL261)	Tumor lysate pulsing (indirect)		[313]
		Melanoma (B16‐OVA)	OVA, αGC	Antigen pulsing (indirect)		[206]
		Melanoma (B16F10)	OVA, Tumor lysates (B16F10)	Tumor lysate pulsing (indirect)		[211]
		Melanoma (B16A2‐Gp100)	Gp100, Mart‐1/Melan‐A	Antigen pulsing (direct acid‐strip)		[314]
		Murine aggressive mastocytoma (P815) Mammary carcinoma (TS/A)	Acid eluted tumor peptides (P815, TS/A)	Antigen pulsing (indirect)		[65]
		Thymoma (EG7, EL4)	OVA	Antigen pulsing (indirect)		[315]
		Thymoma (EG7), Melanoma (BL6‐10)	OVA	Antigen pulsing (indirect)		[204]
		N/A	OVA (full‐length or peptide),	Antigen pulsing (indirect)		[59]
	JAWS II, BM‐DC	Mammary cancer (4T1‐HER2)	CEA‐ECD, HER2‐ECD	Genetic engineering (LA C1C2 domain‐fused antigen expression)		[101]
	PBMC‐derived DC from healthy volunteers	Hepatocellular carcinoma (HepG2, SMMC‐7721)	AFP	Genetic engineering (AFP antigen overexpression)		[316]
	DC from healthy volunteers	Melanoma (K562)	NY‐ESO‐1	Antigen pulsing (direct acid‐strip)		[317]
	BM‐DC, Mo‐DC from healthy volunteers	Melanoma (B16F10)	Melan‐A/MART‐1, Gp100	Antigen pulsing (direct acid‐strip)		[318]
	DEX from BM‐DC vs TEX	Thymoma (EG7), Melanoma lung–metastasis (BL6‐10)	OVA (full‐length or peptide)	Antigen pulsing (indirect), Endogenous (TEX)		[319]
	Hybrid of DEX from BM‐DC and TEX	Head and neck squamous cell carcinoma (SCCVII)	Tumor antigens (SCCVII)	Exogenous (membrane fusion by extrusion, hydrophobic sorption of MPLA (adjuvant))		[117]
	Hybrid of DEX from BM‐DC and iPSC	Melanoma (B16F10), Mesothelioma (AC29), Pancreatic cancer (Pan02)	iPSC‐derived antigens (MART‐1, Gp100, CEA, MUC1)	Exogenous (membrane fusion by extrusion, hydrophobic anchoring of chol–CpG ODN (adjuvant))		[116]
	Hybrid of BM‐DC and GL261 cell	Glioblastoma (GL261)	Tumor antigens (GL261)	Endogenous (PEG‐fused hybrid cells), Exogenous (Electroporation‐mediated loading of cGAMP (STING agonist))		[320]
Tumor derived‐EVs	GL26, primary GBM cell	Glioblastoma	Tumor antigens (GBM)	Endogenous	Phase I/ NCT01550523	[321]
	Malignant ascites	Colorectal cancer	CEA	Endogenous	Phase I	[322]
	Malignant ascites	Gastric cancer	Tumor antigens CEA, CA19‐9, MHC‐I, MHC‐II, Hsp70, Hsp60, Hsp90 identified	Endogenous	Preclinical	[323]
	B16F1 cell	Melanoma (B16F1)	Tumor antigens (B16F1) TRP‐2, Gp100 identified	Endogenous, Genetic engineering (overexpression of CIITA for MHC class II antigen presentation)		[104]
	B16BL6 cell	Melanoma (B16BL6)	Tumor antigens (B16BL6) ‐ Gp100, TRP‐2 identified	Endogenous, Genetic engineering (LA‐fused SAV expression), Exogenous (SAV‐biotin conjugation‐mediated loading of CpG ODN (adjuvant))		[102]
	B16F10 cell	Melanoma, Lung metastasis of melanoma (B16F10)	Tumor antigens (B16F10)	Endogenous		[244]
	CT26 cell	Colorectal cancer (CT26)	Tumor antigens (CT26)	Endogenous		[324]
			Tumor antigens (CT26), MHC class II‐associated tumor antigen	Endogenous, Genetic engineering (overexpression of CIITA for MHC class II antigen presentation)		[105]
	L1210 cell	Leukemia (L1210)	Tumor antigens (L1210)	Endogenous		[325]
			Tumor antigen (L1210) Hsp70, H‐2D (MHC‐I) identified	Endogenous		[326]
		Acute lymphocytic leukemia (L1210)	Tumor antigens (L1210)	Endogenous, Genetic engineering (PD‐L1 shRNA transfected cells)		[239]
	MDA‐MB‐231 cell	TNBC (MDA‐MB‐231 orthotopic; patient organoids)	Tumor antigens (MDA‐MB‐231), α‐LA	Genetic engineering (overexpression of α‐LA immunodominant antigen), Exogenous (electroporation‐mediated loading of Hiltonol (TLR3 agonist), ELANE (ICD inducer))		[110]
	J558 cell	Myeloma (J558)	Tumor antigen (J558), P1A Hsp70 identified	Endogenous, Genetic engineering (overexpression of membrane‐bound Hsp70 (adjuvant))		[238]
			Tumor antigens (J558), P1A ‐ LAMP‐1, AIPI identified	Endogenous, Genetic engineering (overexpression of TNF‐α, IL‐2, IFN‐γ)		[93]
		Plasmacytoma (J558)	Tumor antigens (J558), P1A ‐ IAP (p73), Hsp70 identified	Endogenous		[327]
	RenCa cell	Renal cortical Adenocarcinoma (RenCa)	Tumor antigens (RenCa), G250	Endogenous		[328]
	4T1 cell	Breast cancer(4T1)	Tumor antigens (4T1) ‐ Hsp70 identified	Endogenous, Exogenous (GPI anchoring‐ mediated loading of SEB (adjuvant))		[124]
		Breast cancer (4T1)	Tumor antigens (4T1)	Endogenous		[329]
		TNBC (4T1 orthotopic)	Tumor antigens (4T1), Her2	Endogenous (Her2), Exogenous (electroporation‐mediated loading of CpG, p(I:C) (TLR ligands))		[111]
	A549 cell	NSCLC (A549)	Tumor antigens (A549)	Endogenous, Genetic engineering (overexpression of Rab27a for enhanced exosome release)		[103]
	E.G7‐OVA cell	Thymoma (E.G7‐OVA)	Tumor antigens (E.G7), OVA ‐ Hsp70, Hsp60, Hsc70 identified	Endogenous, Genetic engineering (overexpression of IL‐2)		[92]
	A20 cell	Lymphoma (A20)	Tumor antigens (A20) ‐ Hsp60, Hsp90, MHC I/II, CD86, CD40, RANTES, IL‐1b identified	Endogenous		[330]
	Serum from tumor‐bearing mice (LLC, Hepa1–6 cell)	Lung cancer (LLC)	Tumor antigens (LCC), MUC‐1, GPC3	Endogenous, Exogenous (sonication‐mediated loading of BPQDs (adjuvant/ablation agent for photothermal therapy))		[113]
	Hepa1–6, MC38 cell	Hepatocellular carcinoma (Hepa1–6), Colorectal cancer (MC38)	Tumor antigens (Hela1–6, MC38) ‐ IL‐15 Rα, MHC‐I, Hsp70 identified	Endogenous		[331]
	MCA205, HEK293 cell	Sarcoma (MCA205), Melanoma (B16F10)	SL8	Genetic engineering (SL8 antigen overexpression)		[237]
Macrophage‐derived EVs	RAW264.7 cell	Breast cancer (4T1)	TAA in apoptotic bodies	Antigen pulsing (indirect)	Preclinical	[332]
	Mouse peritoneal, Human PBMC‐derived macrophage fused with tumor nuclei (E.G7, 4T1, B16, MDA‐MB‐231 cell)	Lymphoma (E.G7), Breast cancer (4T1), Melanoma (B16)	Tumor antigens (E.G7, 4T1, B16) ‐ EpCAM, TRP1 identified	Endogenous		[250]
T cell‐derived EVs	Human PBMC‐derived γδ‐T cell	Lymphoma (EBV‐LCL) Gastric carcinoma (SNU‐719)	Tumor lysates (EBV‐LCL) ‐ EBNA1, LMP2a identified	Antigen pulsing (indirect freeze‐thaw)	Preclinical	[333]
Stem cell‐derived EVs	ES‐D3 cell	Lung cancer (LLC), Breast cancer (4T1)	Oncofetal antigens	Endogenous, Genetic engineering (overexpression of GM‐CSF (adjuvant))	Preclinical	[95]
		Metastatic lung cancer (LLC)				[94]
Others	C2C12, SKMC cell	Breast cancer	HER2/neu extracellular domain	Genetic engineering (Nef^mut^‐fused antigen expression)	Preclinical	[334]
	HEK293F cell	Colon carcinoma (CT26‐PAP) Prostate cancer (E6‐PSA)	PSA, PAP	Genetic engineering (LA C1C2 domain‐fused antigen expression)		[100]
	HEK293T cell	N/A	MAGE.A3	Genetic engineering (Nef^mut^‐fused antigen expression)		[335]
		Melanoma (B16‐OVA)	OVA	Genetic engineering (VSV‐G‐fused antigen expression)		[336]
	Serum from mice, Serum from healthy volunteers	Melanoma (B16F10), Colon cancer (MC38), Human Colon cancer (HCT116 spheroid)	Synthetic peptide neoantigens –Trp2, M05, M08, M21, M27, M30, M33, M39 (melanoma) – Adpgk, Reps1, Cpne1, Med12 (colon) – FSP01, FSP02, FSP03 (human colon)	Exogenous (CP05 anchoring)		[127]

a) Direct antigen pulsing refers to incubating EVs with antigens, which are subsequently adsorbed onto MHC class I molecules on the surface of EVs. Indirect antigen pulsing involves adding antigens to the DC culture medium, allowing natural uptake, processing, and presentation via MHC class I or class II pathways.

b) Non‐small cell lung cancer (NSCLC); Bone marrow‐derived dendritic cell (BM‐DC); Monocyte‐derived dendritic cell (Mo‐DC); Ovalbumin (OVA); DSPE and DPPE, phospholipid; Alpha‐fetoprotein (AFP); Carcinoembryonic antigen (CEA); Extracellular domain (ECD); Lactadherin (LA); Peripheral blood mononuclear cells (PBMC); Dendritic cell membrane vesicle (DCMV); Monophosphoryl lipid A (MPLA); Induced pluripotent stem cells (iPSCs); Cholesteryl‐modified CpG oligodeoxynucleotides (chol‐CpG ODN); 2′3′‐cyclic guanosine monophosphate–adenosine monophosphate (cGAMP); Stimulator of interferon genes (STING); Glioblastoma (GBM); Class II transactivator(CIITA); Streptavidin (SAV); Short hairpin RNA (shRNA); Triple‐negative breast cancer (TNBC); α‐Lactalbumin (α‐LA); Toll‐like‐receptor (TLR); Human neutrophil elastase (ELANE); Immunogenic cell death (ICD); Glycosyl‐phosphatidylinositol (GPI); Polyinosinic‐polycytidylic acid (p(I:C)); Black phosphorus quantum dots (BPQDs); PSA (Prostate‐Specific Antigen); PAP (Prostatic Acid Phosphatase); G protein of vesicular stomatitis virus (VSV‐G). Not applicable (N/A).

To enhance immunogenicity and overcome immune tolerance, various cancer vaccine platforms have been developed, including whole‐cell vaccines, peptide/protein‐based formulations, nucleic acid‐based strategies (DNA, mRNA), viral vectors, and DC‐based approaches.^[^ ^184^, ^185^, ^186^ ^]^ Despite the promise of these modalities, only three therapeutic cancer vaccines have thus far gained approval from the U.S. FDA: Bacillus Calmette–Guérin (BCG) for nonmuscle‐invasive bladder cancer, Sipuleucel‐T for metastatic prostate cancer, and Talimogene Laherparepvec (T‐VEC) for advanced melanoma.^[^ ^187^ ^]^ This limited approval highlights the translational challenges faced by therapeutic vaccines, such as safety concerns, complex logistics, and modest clinical efficacy, despite decades of research and technological innovation. Amid these limitations, EV‐based cancer vaccines are now emerging as a highly versatile and promising platform with unique potential for personalized and precision cancer immunotherapy.

### 3.1 DC‐Derived EV Vaccines

#### 3.1.1 Biogenesis and Molecular Characteristics

DCs, as key mediators linking innate and adaptive immunity, are of particular interest in EV‐based vaccine research. Notably, they are known to secrete DEXs (or dexosomes)—constitutively in vitro, regardless of their maturation state.^[^ ^81^, ^188^ ^]^ The quantity and molecular composition of DEX, however, are highly variable and can be significantly influenced by both the maturation stage of the DCs and environmental stimuli.^[^ ^80^ ^]^ Despite such variability, DEX consistently preserve immunological hallmarks reflective of their lineage from professional APCs, thereby supporting their utility as potent immunostimulatory vesicles in therapeutic contexts.

**Figure 2:**
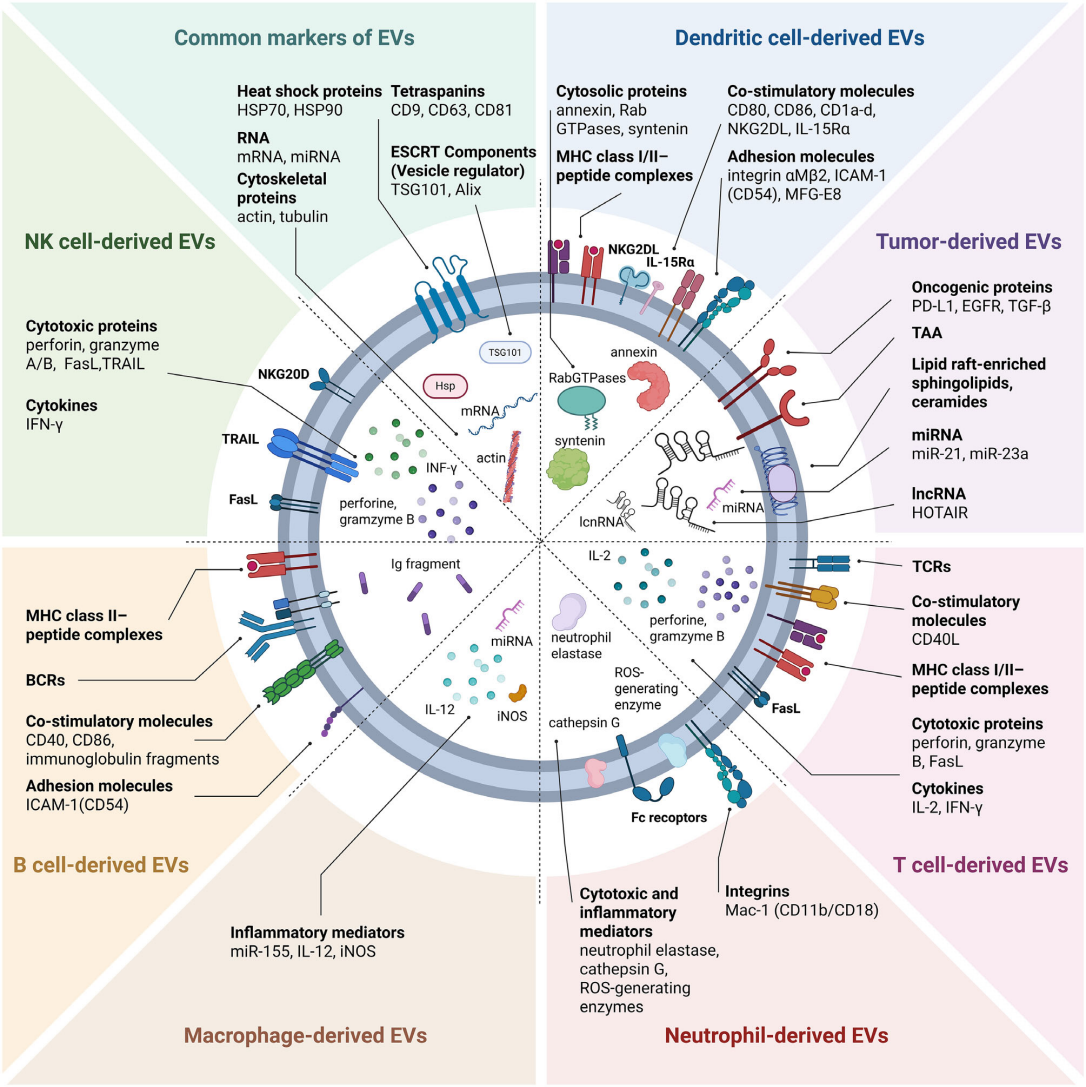
Molecular characterization of EVs derived from distinct immune and tumor cell types EVs ubiquitously express core molecular components, including tetraspanins (CD9, CD63, CD81), heat shock proteins (HSP70, HSP90), various RNAs (mRNA, miRNA), cytoskeletal proteins, and ESCRT‐associated molecules (TSG101, Alix). EVs originating from immune cells are notably enriched with immune‐related molecules, highlighting their pivotal role in intercellular communication and immune modulation. Each molecular constituent reflects the identity of the parental cell, highlighting the cell type‐specific functionality embedded within the EV.

At the molecular level, DEX exhibit hallmark features of professional APCs (**Figure** **2**). Their membranes are enriched with immunostimulatory molecules including MHC class I and II complexes, costimulatory proteins such as CD80 and CD86, and CD1 isoforms (CD1a–d), which collectively support peptide and lipid antigen presentation.^[^ ^43^, ^189^ ^]^ In addition, DEX display adhesion and targeting molecules—such as integrin αMβ2, ICAM‐1 (CD54), and milk fat globule‐EGF factor 8 (MFG‐E8)—that mediate their recognition and uptake by recipient APC, particularly via integrins αvβ3 and αvβ5. Tetraspanin proteins including CD9, CD37, CD63, CD81, and CD82 are abundantly expressed on DEX surface, where they organize functional microdomains critical for intercellular docking and signaling.^[^ ^190^, ^191^ ^]^ To ensure extracellular stability, DEX also incorporate complement regulatory proteins such as CD55 and CD59, which protect DEX from complement‐mediated lysis during circulation. Internally, DEX carry cytoskeletal elements (e.g., actin, tubulin), membrane trafficking regulators (e.g., annexins, RAB GTPases, TSG101), and stress‐response chaperones such as Alix and heat shock proteins HSC73 (HSP70 family) and HSP90.^[^ ^81^ ^]^ These components facilitate EV maturation, antigen processing, and immune activation, reinforcing DEX function as both signaling mediators and immune effectors.

From a structural standpoint, DEXs are also optimized for immune delivery. Their membranes are enriched in sphingomyelin and phosphatidylinositol, which confer increased rigidity and circulatory stability. Moreover, compositional shifts—such as elevated phosphatidylethanolamine and reduced phosphatidylcholine—facilitate membrane fusion and transbilayer lipid movement (“flip‐flop”) with recipient immune cells, thereby enhancing antigen transfer efficiency.^[^ ^192^ ^]^ The presence of phospholipase D2 and phosphatidic acid further supports vesicle‐cell fusion and intracellular delivery.

Beyond their protein and lipid composition, DEX carry a diverse repertoire of RNA molecules, including mRNAs and microRNAs (miRNAs).^[^ ^193^, ^194^ ^]^ These RNAs can be actively transferred to recipient DCs, where they modulate gene expression through post‐transcriptional regulatory mechanisms, contributing to the functional reprogramming of the target DCs. Notably, the phenotypic profile and immunogenic potential of DEX are influenced by the maturation state of donor DCs. Immature DCs typically secrete a larger quantity of DEX, whereas mature DCs tend to generate DEX enriched in immunostimulatory molecules such as MHC class I and II complexes, CD80, CD86, and ICAM‐1, thereby enhancing their capacity to prime robust antigen‐specific T cell responses.^[^ ^81^, ^83^, ^188^, ^195^ ^]^ However, studies have reported conflicting results regarding DEX yield and immunological quality of DEX‐based on the maturation status of donor DCs, underscoring the need for standardized production protocols to ensure reproducibility. Moreover, environmental factors such as exposure to specific cytokines (e.g., IL‐10, IL‐4, IFN‐γ) or cellular stressors (e.g., radiation, senescence) can markedly influence the molecular composition and immunomodulatory functions of DEX. For instance, IFN‐γ‐conditioned DEX exhibit both neuroprotective and immunomodulatory functions through the attenuation of oxidative stress and the reprogramming of microglial activity, thereby broadening their potential immunotherapeutic applications.^[^ ^196^ ^]^

#### 3.1.2 Immunological Functions

**Figure 3:**
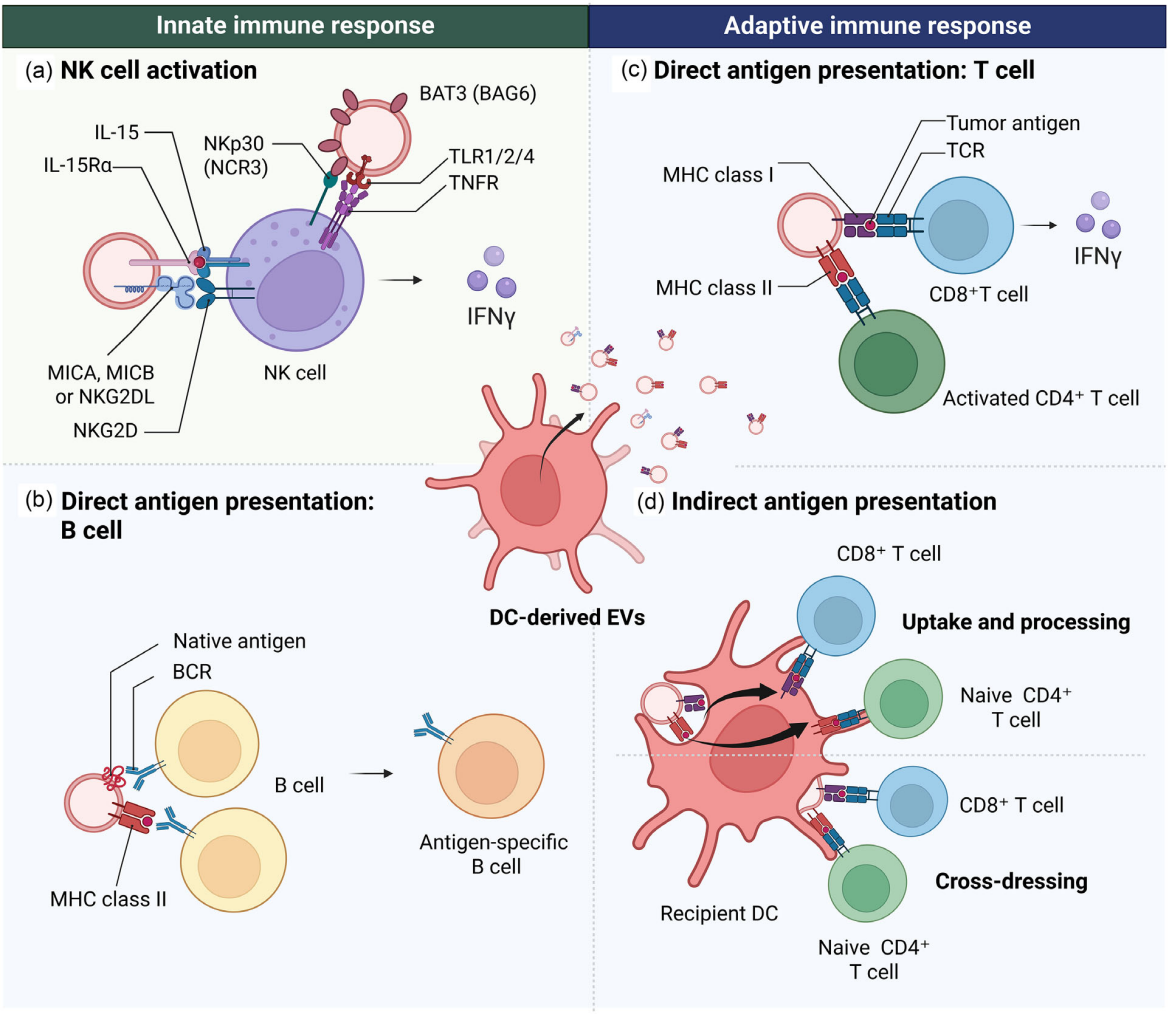
Mechanisms underlying innate and adaptive immune activation via DC–derived EVs. EVs secreted from DCs engage components of both the innate and adaptive immune systems. a) EVs interact with NK cells, triggering their activation and secretion of cytokines such as IFN‐γ b) BCRs on naïve or memory B cells selectively engage with antigen‐presenting EVs, triggering B cell activation, antigen uptake, and subsequent antibody production. c) MHC‐peptide complexes on EVs engage TCRs on CD8^+^ or CD4^+^ T cells, leading to their activation and subsequent proliferation, cytokine secretion, and effector function. d) EVs internalized by APCs initiate indirect T cell priming via two distinct routes: EVs taken up by recipient DCs are processed and present antigen on endogenous MHC molecules, facilitating T cell stimulation. “Cross‐dressing” transfers intact peptide‐MHC complexes to the recipient DC surface via direct fusion of the EV membrane, without requiring additional antigen processing or MHC reloading, allowing rapid T cell engagement.

DEXs exert potent immunomodulatory effects by engaging both innate and adaptive immune systems, making them a highly promising platform in cancer immunotherapy (**Figure** **3**). In the early phase of immune activation, DEXs interact with innate immune cells, particularly natural killer (NK) cells, to enhance antitumor surveillance. They express NK group two member D ligands (NKG2DLs) and interleukin‐15 receptor alpha (IL‐15 Rα), promoting proliferation and IFN‐γ secretion by NK cells.^[^ ^197^, ^198^ ^]^ DEXs also provide ligands such as BAT3/BAG6 for NCR3 and MICA/MICB for NKG2D, directly triggering NK activation.^[^ ^197^, ^199^ ^]^ In murine melanoma models, these molecules supported IL‐15 Rα and NKG2D‐dependent NK cell proliferation and IFN‐γ release, leading to antimetastatic effects. Furthermore, dexosomal TNF ligands interact with TNFRs on NKs to induce cytokine production, and ligands for TLR1/2/4 contribute to TNF expression and NK activation.^[^ ^200^, ^201^ ^]^ These characteristics demonstrate the capacity of DEXs to stimulate NK responses via non‐MHC‐dependent pathways and open new avenues for their use in innate immunity‐based therapies.

In addition to their role in innate immunity, DEXs have been shown to exert powerful effects on adaptive immune activation, particularly through T and B cell engagement. Functionally, DEXs provide several advantages over conventional DC vaccines. They are enriched in antigen–MHC complexes (up to 10–100‐fold higher than DCs), facilitating robust antigen presentation.^[^ ^188^ ^]^ Direct stimulation of the adaptive immune system occurs through MHC class I and II molecules and costimulatory signals (e.g., CD80, CD86) on the surface of DEXs, which can stimulate CD8^+^ and CD4^+^ T cells, respectively. Early evidence by Zitvogel et al. first demonstrated that DEX bearing TAA‐loaded MHC class I complexes could elicit intense CD8^+^ T cell responses and confer tumor protection in immunocompetent mice.^[^ ^65^ ^]^ Notably, these responses were absent in immune‐deficient models, highlighting the requirement for a functional adaptive immune compartment. Coadministration of mature DCs or immunostimulatory adjuvants such as IFN‐γ and Toll‐like receptor (TLR) ligands (e.g., CpG or poly(I:C)) can further enhance the efficacy of DEX‐mediated CD8^+^ T cell activation, in part by augmenting the expression of costimulatory molecules (CD40, CD80, CD86, CD54) on DEXs derived from mature DCs.^[^ ^202^, ^203^ ^]^

However, the activation of naïve CD4^+^ T cells requires capture by bystander DCs.^[^ ^60^ ^]^ DEXs can transfer peptide‐MHC II complexes to MHC II‐deficient DCs, enabling them to activate CD4^+^ T cells.^[^ ^83^ ^]^ DEXs derived from mature DCs are more effective in this process than those from immature DCs.^[^ ^204^ ^]^ Two distinct indirect mechanisms have been proposed to explain how DEXs mediate antigen presentation via DCs. In the first mechanism, DEXs are internalized by bystander DCs.^[^ ^205^ ^]^ Upon internalization, DEX‐derived antigens undergo processing within the endosomal compartment of the recipient DCs. Antigen presentation may occur through two pathways: either by transferring peptide‐MHC complexes from the DEXs onto the DC's endogenous MHC molecules, or by fully reprocessing the internalized antigens for de novo presentation via the host MHC.^[^ ^203^ ^]^ Notably, this mechanism is independent of autologous MHC compatibility. In support of this, Hiltbrunner et al. demonstrated that DEXs devoid of MHC class I were still capable of eliciting OVA‐specific T cell responses and IFN‐γ production, thereby underscoring the feasibility of allogeneic EV‐based immunotherapies.^[^ ^206^ ^]^ In addition, not all DEXs are internalized during this process; a subset remains associated with the DC surface. Immature DCs preferentially internalize DEXs, whereas mature DCs are more inclined to retain them extracellularly.^[^ ^207^ ^]^ Nevertheless, surface‐retained DEXs are also presumed to remain functionally competent in interacting with T cells.

The second mechanism, commonly referred to as “cross‐dressing,” involves the direct transfer of intact peptide‐MHC complexes from the DEX membrane to the plasma membrane of recipient DCs, without the need for internalization or antigen processing.^[^ ^208^ ^]^ This direct molecular transfer enables the recipient DCs to rapidly present antigens and initiate T cell activation. Thery et al. provided compelling in vitro evidence supporting this model by showing that DEXs derived from D1 cells or bone marrow‐derived DCs (BM‐DCs) could activate T cells even when the recipient DCs expressed minimal levels of MHC class II, thus confirming the functional relevance of MHC cross‐dressing in DEX‐mediated immunity.^[^ ^60^ ^]^ Their nanoscale size enables efficient trafficking to secondary lymphoid tissues and interaction with various immune cells via receptor‐mediated mechanisms independent of chemokine gradients.^[^ ^209^ ^]^

#### 3.1.3 Current Strategies and Application

As cell‐free nanovesicles, DEXs inherently lack proliferative, replicative, or differentiation potential, eliminating risks of malignant transformation and underpinning their favorable safety profile.^[^ ^81^ ^]^ They exhibit exceptional physicochemical stability, retaining integrity and bioactivity under cryogenic storage at −80 °C for over six months.^[^ ^210^ ^]^ Additionally, their biogenesis is compatible with GMP^[^ ^188^ ^]^ standards, allowing for scalable and standardized production, a prerequisite for clinical translation. Notably, DEXs demonstrate functional resistance to immunosuppressive cues within the tumor microenvironment, enabling sustained antigen delivery and immune activation even under hostile conditions. These cumulative advantages have spurred a growing body of preclinical and clinical research, with several clinical trials currently underway to validate their efficacy across various cancer types.

Building upon the foundational understanding of DEX biology, several landmark studies have explored bioengineering strategies to enhance their immunotherapeutic potential. Among the earliest and most influential, Besse et al. introduced second‐generation DEXs by stimulating Mo‐DCs from non‐small cell lung cancer (NSCLC) patients with IFN‐γ.^[^ ^86^ ^]^ This maturation process induced the secretion of EVs enriched in immunologically active proteins, including MHC class I, CD86, and BAG6. Functional analyses demonstrated that these IFN‐γ‐conditioned DEXs effectively activated NK cells, evidenced by enhanced degranulation and IFN‐γ production, thereby amplifying antitumor cytotoxicity. Notably, this study provided the first clinical‐grade validation of a DEX‐based immunomodulatory platform, establishing a precedent for immune‐potentiating vesicle design.

While earlier approaches focused on enhancing innate immune engagement, Hiltbrunner et al. implemented a molecular engineering approach to broaden antigenic repertoire and enhance T cell engagement by incorporating full‐length tumor antigens into DEXs.^[^ ^206^ ^]^ This supported concurrent activation of MHC class I and II, effectively bridging CD8^+^ and CD4^+^ T cell responses. Notably, this approach overcame a fundamental limitation of peptide‐only loading and demonstrated that structurally comprehensive antigen delivery via DEXs could significantly elevate adaptive immune activation in autologous settings.

Pursuing the concept of intrinsic immunostimulation, Damo et al. developed a multifunctional DEX platform co‐delivering TAAs and innate immune adjuvants. BM‐DCs were pulsed with OVA peptides in conjunction with poly(I:C), yielding EVs enriched in both antigenic and immunostimulatory components.^[^ ^211^ ^]^ This bioengineered vesicle significantly upregulated maturation marker, MHC class II, on recipient DCs and elicited potent CTL responses in vivo. In B16‐F10 OVA melanoma models, administration of this DEXs led to marked tumor growth inhibition. This strategy demonstrates the feasibility of constructing self‐adjuvanting, cell‐free nanovaccines capable of driving both innate and adaptive immune activation within a single vesicular platform.

Expanding the scope of antigen specificity and therapeutic personalization, Li et al. further advanced DEX bioengineering by developing a neoantigen‐informed vaccine platform tailored to individual tumor profiles.^[^ ^112^ ^]^ By integrating patient‐specific neoepitopes identified through gene sequencing, DCs were pulsed ex vivo to generate DEXs encapsulating tumor‐relevant antigens. In murine models of B16F10 melanoma and MC‐38 colon cancer, these personalized DEXs elicited robust T cell‐ and B cell‐mediated immune responses, resulting in superior tumor control and improved survival outcomes. This study illustrates the versatility of DEXs as modular, programmable vesicles capable of supporting individualized immunotherapy strategies and emphasizes the potential of genomic‐guided antigen selection in maximizing the therapeutic breadth of EV‐based vaccines.

Despite the diversity and sophistication of current DEX engineering strategies, including cytokine‐driven maturation, antigen reprogramming, surface ligand engineering, and neoantigen personalization—relatively few have advanced into clinical trials.^[^ ^86^, ^134^, ^135^, ^197^ ^]^ Moreover, those in clinical evaluation often utilize earlier‐generation constructs with limited functional enhancements.^[^ ^134^ ^]^ Nonetheless, early‐phase clinical data have consistently demonstrated the safety and innate immunoactivity of DEXs, particularly through NK cell engagement, with IFN‐γ–matured DEXs showing enhanced BAG6‐NKp30‐mediated responses.^[^ ^86^ ^]^ Adjunctive strategies such as GM‐CSF co‐delivery and immune preconditioning have offered incremental improvements, while recent preclinical models—including those in hepatocellular carcinoma—underscore the expanding applicability of antigen‐engineered DEXs in modulating the tumor microenvironment.^[^ ^212^ ^]^ These emerging trends suggest that with further optimization in vesicle design, antigen formulation, and combinatorial integration, DEX‐based vaccines are poised to evolve into a clinically viable, cell‐free immunotherapy platform capable of bridging innate and adaptive immunity.

### 3.2 Tumor‐Derived EV Vaccines

#### 3.2.1 Biogenesis and Molecular Characteristics

Tumor‐derived EVs, often referred to as tumor‐derived exosomes (TEXs), have emerged as a functionally distinct EVs, characterized by aberrant molecular profiles and their active participation in cancer progression.^[^ ^213^ ^]^ Unlike EVs secreted by normal cells—which primarily mediate homeostatic intercellular communication—TEXs act as dynamic conveyors of oncogenic signals, modulating immune responses, angiogenesis, stromal remodeling, and premetastatic niches formation.^[^ ^214^, ^215^ ^]^ A hallmark of malignant cells is their elevated rate of EV secretion,^[^ ^216^ ^]^ driven by tumor‐intrinsic metabolic demands and dysregulated endosomal trafficking.^[^ ^217^ ^]^ This increase in TEX output enhances intercellular signaling within the TME, promoting immune evasion and metastatic dissemination.^[^ ^218^ ^]^ By persistently exporting oncogenic cues that shape both local and systemic environments in favor of malignancy, cancer cells utilize TEXs as strategic tools to exert molecular control over their microenvironment.

On the molecular level, TEX biogenesis is influenced by oncogenic mutations (e.g., KRAS, EGFR, p53), and tumor‐specific microenvironmental stressors such as hypoxia, acidosis, and inflammation.^[^ ^219^ ^]^ Hypoxia‐inducible factor‐1α (HIF‐1α) upregulates key components of endosomal sorting complex required for transport (ESCRT) pathway (e.g., TSG101, CHMP4) and Rab GTPases (Rab27a/b), facilitating MVB maturation and EV release. Final EV release is governed by coordinated action of Rab GTPases and SNARE proteins, including Rab27a/b, VAMP7, and syntaxin 6, which are frequently upregulated in cancer through RAS/ERK and EGFR signaling.^[^ ^219^, ^220^ ^]^ Furthermore, the adaptor protein Alix regulates ESCRT‐III recruitment and cargo loading, while also interacting with the syndecan–syntenin axis;^[^ ^221^ ^]^ its depletion disrupts PD‐L1 trafficking and amplifies immune suppression.^[^ ^222^ ^]^ ESCRT‐independent mechanisms, such as ceramide‐driven budding via nSMase2 and CD63‐mediated membrane invagination, also contribute to TEX release under stress conditions.^[^ ^223^, ^224^ ^]^ TEX cargo is selectively enriched in oncogenic proteins (e.g., EGFR, PD‐L1, TGF‐β), TAAs, heat shock proteins, and noncoding RNAs (e.g., miR‐21, HOTAIR), which reprogram recipient cells to support tumor growth and immune escape (Figure 2).^[^ ^225^ ^]^ Their lipid composition—particularly high levels of sphingolipids and ceramides—enhances membrane rigidity and facilitates lipid raft‐mediated cell targeting.^[^ ^226^ ^]^

Together, the oncogenic reprogramming of biogenesis, coupled with selective cargo loading and immunosuppressive features, highlights the complex role of TEXs in both promoting tumor progression and serving as potential platforms for cancer immunotherapy and diagnostic applications. A deeper mechanistic understanding of TEX biology will be essential to accurately assess their translational potential in cancer vaccines and immunotherapy.

#### 3.2.2 Immunological Functions

Unlike EVs derived from healthy cells, TEXs carry a distinct molecular signature, as described in the previous section. These vesicles participate in bidirectional crosstalk between tumor and immune cells, mediating both immunosuppressive and immunostimulatory signals that influence cancer progression. Depending on the context, TEXs may either facilitate immune escape^[^ ^227^ ^]^ or contribute to the activation of tumor‐specific immune responses.^[^ ^228^ ^]^

Within the innate immune compartment, TEXs impair NK cell cytotoxicity by downregulating key activation markers such as NKG2D and CD69 through inhibition of the STAT5 and JAK3 signaling pathways.^[^ ^229^ ^]^ TEXs also suppress perforin secretion and promote the accumulation of myeloid‐derived suppressor cells (MDSCs), thereby weakening NK‐mediated surveillance. Additionally, TEXs activate the TGF‐β/SMAD signaling axis, suppressing IL‐2‐induced NK cytotoxicity and reducing peripheral NK populations. Paradoxically, TEXs expressing surface HSP70 have been shown to enhance NK activation via granzyme B release and upregulation of activating receptors, highlighting their regulatory role.

TEXs also affect macrophage polarization, favoring the tumor‐promoting M2 phenotype.^[^ ^230^ ^]^ Through TLR/NF‐κB signaling, TEXs induce the secretion of proinflammatory cytokines such as IL‐6, TNF‐α, and CCL2, supporting tumor‐associated macrophage survival. Moreover, Wnt5a upregulation in macrophages by TEXs enhances β‐catenin‐independent signaling in tumor cells, promoting invasiveness.

In DCs, TEXs exhibit both inhibitory and stimulatory effects. Immunosuppressive factors such as TGF‐β, HSP72, and prostaglandin E2 within TEXs impair DC differentiation and maturation by activating STAT3 and downregulating costimulatory molecules.^[^ ^231^ ^]^ TEXs also induce IL‐6 phosphorylation and reduce MHC class II and TLR4 expression, driving DCs toward a tolerogenic phenotype.^[^ ^232^ ^]^ Conversely, DCs can internalize TEXs and present tumor‐derived antigens, leading to upregulation of MHC and costimulatory receptors (CD80, CD86), secretion of immunostimulatory cytokines, and T helper cell activation under appropriate conditions.^[^ ^233^ ^]^

TEXs exert various immunosuppressive effects on T cells. They inhibit CD8^+^ T cell proliferation, induce apoptosis via Fas and PD‐1 signaling, and reduce IL‐2 responsiveness.^[^ ^227^ ^]^ TEXs also reprogram CD4^+^ and CD8^+^ T cells toward exhausted or regulatory phenotypes via expression of CD39, CD73, Galectin‐1, and TGF‐β1. Furthermore, they promote the expansion of FOXP3^+^ regulatory T cells directly or through the induction of tolerogenic DCs.^[^ ^234^ ^]^ Despite these suppressive roles, TEXs retain the capacity to present antigens through surface MHC class I and II complexes and can potentially prime antigen‐specific T cells, particularly memory CTLs.

Although the effects of TEXs on B cells are less well‐characterized, some studies suggest that TEXs can drive naïve B cells toward a regulatory phenotype and interfere with antibody‐dependent cytotoxicity.^[^ ^235^ ^]^ In contrast, other studies have suggested that TEXs might enhance antibody production, although the underlying mechanisms remain poorly understood.

#### 3.2.3 Current Strategies and Application

TEXs have emerged as dynamic immunological entities with substantial potential in cancer vaccine development. Unlike their unmodified counterparts, bioengineered TEXs can be strategically modified to overcome their inherent immunosuppressive properties and to enhance tumor‐specific immune responses. Recent advances in TEX‐based vaccine platforms illustrate how tailored molecular and immunological modifications can transform TEXs into potent activators of adaptive immunity.^[^ ^236^ ^]^

In recent studies, researchers have systematically engineered TEXs into immunostimulatory platforms through four complementary engineering strategies: 1) increasing antigenic payloads—either by direct overexpression of TAAs^[^ ^237^ ^]^ or by enhancing EV secretion^[^ ^103^ ^]^ and MHC expression pathways;^[^ ^104^, ^105^ ^]^ 2) priming innate immune responses via incorporation of TLR agonists, immunogenic cell death (ICD) inducers,^[^ ^110^ ^]^ CpG oligodeoxynucleotides,^[^ ^102^ ^]^ or HSP70;^[^ ^238^ ^]^ 3) promoting T cell activation through the display of superantigens^[^ ^124^ ^]^ or suppression of PD‐L1 expression;^[^ ^239^ ^]^ and 4) modulating cytokine profiles by enriching vesicles with proinflammatory mediators such as TNF‐α, IL‐2, and IFN‐γ.^[^ ^93^ ^]^ One of the most notable strategies was demonstrated by Shi et al., who investigated the effects of IFN‐γ fusion protein‐surface anchored TEXs derived from RM‐1 prostate cancer cells.^[^ ^240^ ^]^ Streptavidin (SA)‐tagged IFN‐γ was efficiently immobilized onto the surface of biotinylated EVs via protein‐anchoring strategy. The resulting IFN‐γ‐TEX vaccine significantly increased F4/80^+^CD86^+^ M1 macrophages in tumor tissues reflecting immunological effects driven by IFN‐γ signaling. Furthermore, in vivo administration of IFN‐γ‐TEX vaccine led to a marked reduction in FOXP3^+^ regulatory T cells and elevated IFN‐γ production by CD8^+^ T cells, indicating a reprogramming of TEX function from immunosuppressive to immune‐activating. This study established a surface modification of TEXs with immunostimulatory molecules like IFN‐γ for reconfiguring its immunogenicity. Building upon this, Zuo et al. developed a genetically engineered fusion protein by linking the damage‐associated molecular pattern (DAMP) molecule HMGN1 to the EV‐anchoring peptide CP05.^[^ ^241^ ^]^ TEXs secreted from Hepa1–6 hepatocellular carcinoma cells, bearing HMGN1 (TEX‐N1ND), were pulsed onto DCs to generate the DC‐TEX‐N1ND vaccine. Due to the TEX‐N1ND‐triggered DC activation, this construct elicited potent CD8^+^ T cell responses and significantly delayed tumor progression in vivo. By codelivering endogenous tumor antigens and exogenous immunostimulatory signals including DAMPs within a single vesicle, this platform elicited robust immune activation and expanded the functional scope of TEX‐based vaccines. In a similar approach, Morishita et al. designed TEX were transfected with a plasmid encoding streptavidin–lactadherin (SAV–LA) fusion protein, enabling its display on the exosomal surface.^[^ ^102^ ^]^ This modification allowed for the stable attachment of biotinylated CpG DNA, thereby facilitating the co‐delivery of endogenous tumor antigens and TLR9 agonists to DCs. Upon uptake by DCs, CpG DNA‐modified EVs promoted cellular activation, which in turn enhanced antigen cross‐presentation, upregulated secretion of IL‐12p40 and TNF‐α, ultimately driving a potent induction of CD8^+^ T cell responses.

Expanding upon prior surface‐modification strategies, Huang et al. engineered breast cancer–derived TEXs (HELA‐Exos)—a TEX vaccine platform derived from α‐lactalbumin–overexpressing breast cancer cells—electroporated to load the TLR3 agonist Hiltonol and the ICD inducer human neutrophil elastase (ELANE).^[^ ^110^ ^]^ Upon systemic administration, HELA‐Exos demonstrated efficient tumor targeting, promoted CD8^+^ T cell–mediated cytotoxicity, and suppressed tumor progression in both orthotopic triple negative breast cancer (TNBC) models and patient‐derived tumor organoids.

While previous strategies predominantly relied on extrinsic modification of TEXs through protein anchoring or physical cargo loading, emerging studies have introduced a novel paradigm centered on intrinsic reprogramming of TEXs via engineering the molecular profile of their parental cell. One such example is the work by Huang et al., who employed lentiviral‐mediated small hairpin RNA (shRNA) to silence PD‐L1 expression in acute lymphoblastic leukemia cells.^[^ ^239^ ^]^ This genetic intervention led to a marked reduction of exosomal PD‐L1, thereby alleviating the immunosuppressive effects of leukemia‐derived exosomes (LEXs). The designed vesicles enhanced DC maturation, promoted Th1 cytokine secretion, and elicited antigen‐specific CTL responses both in vitro and in vivo. Building on this conceptual framework, Han et al. introduced a more comprehensive paradigm of intrinsic TEX reprogramming through pharmacological modulation.^[^ ^242^ ^]^ By treating tumor cells with Verteporfin—a clinically approved inhibitor of YAP signaling—they concurrently attenuated pro‐tumorigenic traits by disrupting YAP–TEAD interactions and promoting YAP degradation in the cytosol, which led to reduced tumor cell proliferation and migration, as reflected by the downregulation of proliferation‐associated genes (e.g., CDC5L, CDKN2AIPNL, CDC37, CDC27) and migration‐related genes (e.g., ITGA7, ITFG2, CDH26, MYO18A), while enhancing the immunogenicity of secreted TEXs. This modulation was achieved through suppression of autophagy and induction of ICD, resulting in the accumulation of cytosolic dsDNA, calreticulin, HMGB1, and HSP70 within TEXs. These attenuated and immunogenic TEXs (AI‐TEXs) robustly activated the cGAS–STING pathway in DCs, promoted antigen cross‐presentation, and elicited potent CD8^+^ T cell‐mediated responses in both preventive and therapeutic tumor models.

These cumulative advances in TEX bioengineering have driven diverse preclinical strategies to overcome their intrinsic immunosuppressive properties and enhance antitumor immunity. Versatile application platforms—including ICD–derived vesicles,^[^ ^243^ ^]^ and microneedle‐based delivery systems^[^ ^244^ ^]^—have emerged to address safety, scalability, and clinical practicality. Owing to their autologous origin, high abundance of TAAs, and intrinsic compatibility with APCs, TEXs offer a uniquely adaptable platform for personalized immunotherapy. They can be directly isolated from patient‐derived sources including sera, malignant effusions, or tumor‐conditioned media, allowing individualized vaccine production without the need for synthetic antigen design. Recent engineering efforts further position TEXs at the forefront of immunotherapy by integrating structural design, antigen specificity, and systemic immune modulation into a single platform.

### 3.3 Other Cell‐Derived Vaccine

#### 3.3.1 Macrophage‐Derived EVs

Macrophage‐derived EVs, particularly those secreted by M1‐polarized macrophages (M1‐Exos), are secreted as a consequence of classical macrophage activation, typically initiated by exposure to IFN‐γ or lipopolysaccharide (LPS).^[^ ^245^ ^]^ These EVs are molecularly enriched with inflammatory mediators such as miR‐155, interleukin‐12 (IL‐12), inducible nitric oxide synthase (iNOS), and other immunostimulatory components—including proinflammatory cytokines, costimulatory molecules, and microRNAs—that collectively reflect the functional phenotype of their parental cells and potentiate antigen presentation and T cell activation (Figure 2).^[^ ^246^ ^]^ Conversely, EVs derived from M2‐polarized macrophages are typically enriched in immunosuppressive factors such as miR‐21 and TGF‐β, which may contribute to immune tolerance or the induction of regulatory T cells.^[^ ^247^ ^]^ This immunological dichotomy underscores the significance of macrophage polarization in dictating EV cargo and highlights the potential to harness such vesicles as tunable immune modulators in vaccine platforms.

Functionally, M1‐Exos exert a profound immunoregulatory influence within the TME. These vesicles have been shown to convert tumor‐associated macrophages (TAMs) from an M2‐like, immunosuppressive phenotype into an M1‐like, proinflammatory state.^[^ ^246^ ^]^ This repolarization is mechanistically associated with the induction of TNF‐α, IL‐6, and nitric oxide synthesis, reshaping the TME into a milieu that favors cytotoxic immune cell infiltration. Concurrently, M1‐Exos stimulate DC maturation and enhance CD8^+^ T lymphocyte activation.^[^ ^248^ ^]^ While direct evidence for Th1 polarization is limited, their proinflammatory cargo suggests a potential role in supporting CD4^+^ T cell differentiation toward effector phenotypes in an inflammatory niche.^[^ ^245^ ^]^ These concerted effects allow M1‐Exos to bridge the innate and adaptive immune compartments in a temporally and spatially coordinated manner.

Beyond their intrinsic immunomodulatory roles, M1‐Exos have garnered interest as emerging platforms for cancer vaccine development. In preclinical models, M1‐Exos loaded with tumor antigens or immunogenic peptides have been shown to enhance antigen cross‐presentation, activate APCs, and amplify CTL responses.^[^ ^249^ ^]^ Importantly, their inflammatory context provides endogenous adjuvanticity, supporting T cell priming without the need for exogenous adjuvants. In 4T1 breast cancer, M1‐Exos have been shown to reprogram TAMs toward inflammatory phenotypes and stimulate proinflammatory cytokine production (e.g., IL‐6, IL‐12, iNOS).^[^ ^114^ ^]^ More recent innovations have introduced hybrid vesicles engineered by fusing membranes from M1‐polarized macrophages and tumor cells—referred to as artificial macrophage–tumor exosomes (aMT‐Exos).^[^ ^250^ ^]^ These chimeric vesicles exhibit improved lymph node homing and tumor site accumulation, thereby enhancing the delivery and contextualization of antigens in immunologically relevant microenvironments.

Rather than functioning solely as adjuvants or antigen carriers, M1‐Exos uniquely integrate immunostimulation, macrophage reprogramming, and T cell activation—an intersectional role that sets them apart among vesicle‐based cancer vaccines.^[^ ^249^ ^]^ This distinctive combination of immune reprogramming and antigen delivery positions M1‐Exos not only as therapeutic vectors but also as dynamic regulators of the tumor immune landscape, paving the way for their strategic inclusion in multimodal immunotherapy frameworks.

#### 3.3.2 T Cell‐Derived EVs

Upon antigen recognition and TCR engagement, T cells position MVBs toward the contacting site with APC enabling the focused release of immune‐active EVs.^[^ ^251^, ^252^ ^]^ This process is critically dependent on the MAL protein, which functions as a spatial organizer that integrates tetraspanin‐enriched microdomains with ceramide‐rich nanodomains, facilitating the formation of intraluminal vesicles destined for EV release.^[^ ^253^ ^]^ In addition to TCR engagement, cytokines such as interleukin‐2 (IL‐2) and IL‐12 further modulate vesicle release dynamics, thereby fine‐tuning EV secretion in an activation‐ and subset‐specific manner.^[^ ^254^, ^255^ ^]^

T cell‐derived EVs (T cell‐derived exosomes, TExos) encapsulate a repertoire of immune‐regulatory cargo that mirrors the effector status of their parent cells, including cytotoxic proteins (e.g., perforin, granzyme B), cytokines (e.g., IL‐2, IFN‐γ), costimulatory ligands (e.g., CD40L), and MHC‐peptide complexes (Figure 2).^[^ ^256^, ^257^ ^]^ While the delivery of such cargo to surrounding immune cells via paracrine or autocrine communication is a shared feature of many immune‐derived EVs, TExos possess several distinguishing characteristics. Beyond those, TExos play an active role in immune coordination by delivering immune‐active signals that promote DC cross‐priming, which enhances secondary CD8^+^ T cell activation and systemic antitumor immunity.^[^ ^256^ ^]^ Notably, they preserve the antigen specificity of their parental TCRs, enabling targeted immunological signaling.^[^ ^255^, ^258^, ^259^, ^260^ ^]^ These features underscore their relevance not only as immunological messengers but as controllable and safe delivery platforms in therapeutic contexts.

Among T cell subsets, EVs derived from CD8^+^ CTLs (CD8^+^ TExos) are functionally specialized to execute direct tumor cell killing.^[^ ^256^ ^]^ Upon antigenic stimulation, CD8^+^ T cells secrete EVs enriched with cytotoxic effector proteins including perforin, granzyme B, and Fas ligand (FasL), which collectively induce both caspase‐dependent and extrinsic apoptotic pathways in target tumor cells.^[^ ^261^ ^]^ These vesicles may partially reflect the antigen specificity of their parent T cells and selectively target tumor cells expressing cognate antigens, thereby minimizing off‐target effects. Beyond their direct cytotoxicity, accumulating evidence indicates that CD8^+^ TExos contribute to remodeling the tumor microenvironment, enhancing antitumor immunity in coordination with other immune components.^[^ ^256^ ^]^ Their ability to synergize direct cytotoxicity with secondary immune amplification renders them attractive agents for combination with checkpoint inhibitors or neoantigen‐targeted strategies.

CD4^+^ T cell‐derived EVs (CD4^+^ TExos) are secreted upon TCR stimulation during immunological synapse formation and are enriched with MHC class II molecules,^[^ ^262^ ^]^ CD40 ligand (CD40L), and other costimulatory signals essential for effective T cell help.^[^ ^263^, ^264^ ^]^ Functionally, CD4^+^ TExos have been shown to enhance B cell activation and antibody class switching by engaging CD40 on antigen‐presenting B cells, thereby mimicking the role of classical T helper cells.^[^ ^263^ ^]^ Engineered CD4^+^ TExos further amplify immune modulation through surface tethering of IL‐2, a strategy shown to restore effector function in exhausted CD8^+^ T cells and attenuate immunosuppressive signaling via PD‐L1 in melanoma cells.^[^ ^265^ ^]^ These findings validate CD4^+^ TExos as modulators of humoral and cellular immunity and expand their immunological role beyond canonical T cell helper activity.

Chimeric antigen receptor (CAR) T cell‐derived EVs (CAR‐Exos) represent a novel modality in adoptive immunotherapy, combining the precision of engineered antigen recognition with the scalable and cell‐free advantages of EVs. Upon antigen engagement and immune synapse formation, CAR‐T cells secrete EVs bearing functional CARs and cytotoxic proteins such as granzyme B and perforin.^[^ ^258^, ^266^ ^]^ These vesicles preserve the tumor antigen specificity and cytotoxic activity of their parental CAR‐T cells. In preclinical tumor models, CAR‐Exos targeting antigens such as HER2, EGFR, and mesothelin have exhibited potent antitumor efficacy, inducing apoptosis in tumor cells and significantly inhibiting tumor growth without observable CRS, a frequent complication of cellular therapies.^[^ ^258^, ^266^ ^]^ Mechanistically, CAR‐Exos can also influence the tumor microenvironment by downregulating immunosuppressive cells and triggering proinflammatory cytokine cascades. Their nanoscale properties and biological stability further support their development as off‐the‐shelf nanotherapeutics capable of repeated systemic administration.^[^ ^267^ ^]^ These advantages collectively position CAR‐Exos as a convergent platform that fuses the precision of cell therapy with the safety and versatility of vesicle‐based delivery.

TExos, spanning natural and engineered subsets, offer a modular, programmable platform that preserves the specificity and functionality of their parental T cells, enabling precision‐guided immune activation for cancer immunotherapy.

#### 3.3.3 B Cell‐Derived EVs

B cell‐derived EVs (B cell‐derived exosomes, BExos) constitute a unique subset of immune‐regulatory vesicles that are secreted upon B cell activation, particularly in response to T cell‐dependent signals involving B cell receptor (BCR) ligation and CD40 engagement.^[^ ^268^ ^]^ BExos are molecularly distinguished by the presence of MHC class II–peptide complexes and costimulatory molecules such as CD40 and CD86, which facilitate their interaction with T cells (Figure 2).^[^ ^18^ ^]^ Importantly, BExos display surface‐associated BCR complexes along with a functionally active pool of monomeric immunoglobulin M (IgM), present both on the membrane and within the vesicle lumen.^[^ ^269^ ^]^ This IgM retains antigen specificity and functions independently from the pentameric form of IgM secreted via classical secretory pathways. This combination of antigen presentation through MHC II and antibody‐mediated recognition allows BExos to engage target cells through multiple immunological interfaces. Given these properties and the expression of costimulatory molecules, BExos possess intrinsic properties advantageous for vaccine design—namely, endogenous adjuvanticity, cell‐specific targeting, and efficient antigen presentation.^[^ ^18^ ^]^ Quantitative proteomic analyses of purified BExos have further revealed selective enrichment of molecules such as CD20, HSP70, and 14‐3‐3ε, many of which are known to associate with antigen processing or membrane trafficking machinery.^[^ ^270^ ^]^ Moreover, the composition and release of BExos are dynamically regulated by cognate CD4^+^ T cell engagement, which enhances EV secretion and facilitates the preservation of peptide‐loaded MHC II complexes otherwise destined for lysosomal degradation.^[^ ^85^ ^]^

Functionally, BExos have been shown to initiate antigen‐specific CD4^+^ T cell activation and proliferation.^[^ ^85^ ^]^ In an allergic context, BExos loaded with birch allergen peptide (Bet v 1), induced robust proliferation of allergen‐specific T cell lines and stimulated the production of IL‐5 and IL‐13—signatures of Th2 polarization.^[^ ^41^ ^]^ This capacity for direct T cell stimulation by BExos underscores their potential to act as autonomous antigen‐presenting nanostructures. Building on these findings, recent studies have also explored the use of BExos as a vaccine delivery platform in cancer immunotherapy. Additionally, Chen et al. demonstrated that EVs derived from diffuse large B‐cell lymphoma could effectively prime DCs, which, when transferred in vivo, induced potent cytotoxic T cell responses, suppressed regulatory T cells, and elicited Th1‐dominant cytokine production.^[^ ^271^ ^]^ Notably, these outcomes were superior to those induced by conventional tumor lysates, suggesting the superior immunogenicity of BExos. By leveraging their intrinsic antigen‐presenting capability and antibody‐mediated targeting, BExos activate multiple immune pathways—bridging humoral recognition with cell‐mediated cytotoxicity—and thereby establish themselves as a versatile platform for vesicle‐based cancer vaccines.^[^ ^272^ ^]^

#### 3.3.4 NK Cell‐Derived EVs

NK cell‐derived EVs (NK cell‐derived exosomes, NK‐Exos) have demonstrated distinct cytotoxic potential against malignancies, primarily through the vesicular delivery of NK‐specific effector proteins (Figure 2).^[^ ^273^ ^]^ These EVs selectively incorporate and export perforin, granzyme B, FasL, and TRAIL—molecules that closely mirror the cytolytic arsenal of their parental NK cells and mediate direct apoptosis of diverse tumor targets via both intrinsic and extrinsic apoptotic pathways.^[^ ^274^, ^275^, ^276^, ^277^ ^]^

Preclinical studies have highlighted the antitumor activity of NK‐Exos in selected cancer models, such as melanoma and breast cancer. In murine models of melanoma, administration of NK‐92 cell‐derived EVs led to significant tumor regression, coinciding with FasL‐ and TNF‐α‐mediated induction of apoptosis in B16F10 cells.^[^ ^273^ ^]^ Comparable effects have been observed in breast cancer models,^[^ ^278^ ^]^ where paclitaxel‐loaded NK‐Exos enhanced apoptosis in MCF‐7 cells via upregulation of Bax and caspase‐3. Extending these observations, recent work has demonstrated the efficacy of NK‐Exos in neuroblastoma.^[^ ^279^ ^]^ In MYCN‐amplified neuroblastoma models, NK‐Exos enriched with miR‐186 suppressed tumor proliferation and migration by targeting MYCN, AURKA, and TGF‐β receptors. Notably, they also overcame TGF‐β–mediated suppression of NK cell cytotoxicity, thereby restoring innate immune function within the tumor microenvironment.

Furthermore, the cytotoxic potency of NK‐Exos can be enhanced through cytokine‐driven programming of the donor cells. Enomoto et al. demonstrated that NK‐92 cells primed with IL‐15 and IL‐21 produced EVs with elevated levels of granzyme B and CD226.^[^ ^280^ ^]^ These modifications not only amplified direct cytolytic activity but also promoted enhanced vesicle uptake via macropinocytosis, indicating that both functional and biophysical parameters of NK‐Exos are amenable to external modulation.

Expanding their therapeutic versatility, NK‐Exos have also been harnessed as delivery vehicles for chemotherapeutic agents. Doxorubicin‐loaded NK‐Exos (NK‐Exos‐Dox) elicited robust apoptotic signaling in human breast carcinoma cells supporting their utility as both innate immune effectors and modular nanocarriers.^[^ ^281^ ^]^ Also, NK‐Exos have been successfully engineered to deliver therapeutic siRNA payloads. In a representative study, NK92MI cells were modified to load BCL‐2 targeting siRNAs into NK‐Exos, which upon uptake by ER^+^ breast cancer cells, led to potent downregulation of BCL‐2 expression.^[^ ^282^ ^]^ This gene silencing effect triggered mitochondrial membrane disruption, caspase‐3/7 and caspase‐9 activation, and significantly enhanced apoptosis in tumor cells, while sparing nonmalignant counterparts.

These findings position NK‐Exos as vesicular extensions of innate immunity that can simultaneously mediate immune activation and deliver therapeutic payloads, highlighting their potential for integrated immuno‐chemotherapeutic strategies against tumors resistant to conventional adaptive immune therapies.

#### 3.3.5 Neutrophil‐Derived EVs

Neutrophil‐derived EVs (Neutrophil‐derived exosomes, NExos) are released predominantly during early neutrophil activation, particularly in the context of NETosis and inflammatory degranulation.^[^ ^283^ ^]^ NExos uniquely reflect the proteolytic and oxidative arsenal of their parental neutrophils, encapsulating granule‐derived effectors such as neutrophil elastase (NE), cathepsin G, proteinase 3 (PR3), and enzymes responsible for the generation of reactive oxygen species (ROS) (Figure 2).^[^ ^283^ ^]^ The surface expression of integrins such as CD11b/CD18 (Mac‐1) further facilitates their preferential localization to sites of vascular inflammation, positioning them as active participants in early immune orchestration within the tumor microenvironment.^[^ ^283^, ^284^ ^]^

NExos, particularly those derived from N1‐polarized neutrophils, have been reported to exert immunomodulatory effects by promoting macrophage activation and T cell proliferation through the transfer of proinflammatory mediators.^[^ ^284^ ^]^ These NExos carry molecules such as IL‐1β, IL‐2, and IL‐4, which may contribute to shaping a more immunostimulatory tumor microenvironment. Addition to this immunostimulatory role, Zhang et al. demonstrated NExos possess inherent cytotoxic properties capable of inducing apoptosis in tumor cells via caspase activation and delivery of effector molecules such as FasL and perforin.^[^ ^285^ ^]^ Furthermore, bioengineered NExos with SPIONs enable magnetic targeting and enhanced tumor accumulation, improving their therapeutic precision and efficacy.

## 4 Challenges and Perspectives of EV‐Based Vaccines

As previously outlined, bioengineered EV‐based vaccines represent a promising frontier in immunotherapy, yet their clinical translation remains hindered by multifaceted challenges. While prior sections detailed various cellular sources, antigen loading techniques, and delivery routes, a critical analysis of engineering hurdles, clinical translation limitation, and strategic opportunities is essential to contextualize future applications. Here, we discuss the major obstacles that must be addressed to advance EV vaccines toward widespread clinical use.

A fundamental challenge in EV vaccine development is the scalability of production. Among various cellular sources, DEX are particularly well characterized and exhibit favorable immunostimulatory properties, as they display antigen‐loaded MHC, costimulatory molecules, and NK cell‐activating signals while lacking immunosuppressive cargo. However, their low yield and high cost hinder further development of large‐scale application, although engineering approaches such as enhanced EV biogenesis pathways and large‐scale DC culture systems have been explored to improve scalability.^[^ ^286^, ^287^ ^]^ Alternative sources such as tumor cells or immortalized epithelial cell lines offer higher productivity,^[^ ^288^ ^]^ yet raise regulatory and safety concerns—tumor cells^[^ ^289^, ^290^, ^291^ ^]^ due to their oncogenic origin and the risk of immunosuppressive or tumor‐promoting EV cargo, and epithelial lines^[^ ^292^, ^293^ ^]^ owing to potential genomic alterations and uncertain long‐term safety, both of which complicate GMP compliance and translational applicability.

Another unresolved issue is the lack of standardization across EV production, isolation, and characterization workflows. Although guidelines such as MISEV provide a framework for minimal experimental standards, implementation remains inconsistent across research groups, particularly in EV isolation techniques, quantification methods, and marker characterization. This variability further complicates the reproducibility of findings, limits cross‐study comparisons, and ultimately reduces the reliability of EV‐based platforms for clinical translation.

In addition to inconsistent experimental practices, EVs exhibit notable heterogeneity due to their composite nature, consisting of diverse proteins, lipids, and nucleic acids.^[^ ^294^ ^]^ Unlike monocomponent nanoparticles such as antibodies, recombinant proteins, EVs inherently reflect the biological complexity of their donor cells, making their physical properties highly sensitive to isolation methods and environmental context. Reflecting this, recent consensus guidelines have underscored the necessity of reporting purification protocols, as accumulating evidence indicates that key biophysical parameters—such as size distribution, zeta potential, and protein‐to‐lipid ratios—are substantially influenced by the isolation strategy. For instance, Caponnetto et al. reported that polymer‐based precipitation yielded smaller particles than ultracentrifugation, which in turn led to more rapid cellular uptake and enhanced cell motility—highlighting how purification‐dependent size variation can directly modulate therapeutic performance.^[^ ^155^ ^]^

To translate this biological complexity into reproducible and clinically viable products, more stringent quality control measures are essential—particularly in the context of GMP‐compliant manufacturing.^[^ ^295^, ^296^ ^]^ In response to the growing clinical demand, scalable production systems must incorporate well‐defined upstream and downstream processes tailored to the EV source, each offering distinct advantages in yield, safety, and scalability. Regardless of the origin, consistent product quality relies on the integration of advanced purification methods—such as TFF, affinity chromatography, and field‐flow fractionation—alongside standardized storage conditions remain critical to maintaining batch‐to‐batch reproducibility and clinical‐grade quality. In fact, advanced engineering strategies such as microfluidic systems^[^ ^297^ ^]^ and machine learning (ML)^[^ ^298^ ^]^ offer new tools for quality control and standardization of EV‐based therapeutics. Microfluidic platforms, as previously described in section 2.2.2, enable an all‐in‐one workflow that integrates EV capture, molecular cargo loading, and release within a single system—minimizing manual handling, enhancing product consistency, and supporting scalable, GMP‐compatible manufacturing. Complementarily, the integration of electrokinetic profiling with quantum ML models has been used to distinguish EVs from nanoparticles with high precision, offering a promising approach toward the standardization of EV formulations and improved product consistency.

In terms of storage and distribution, lyophilization has emerged as a practical approach for preserving EV stability given that repeated freeze‐thaw cycles have been shown to cause significant structural and functional degradation of EVs, including loss of RNA cargo, increased vesicle size, and reduced bioactivity.^[^ ^299^, ^300^, ^301^ ^]^ However, data regarding long‐term bioactivity and cargo integrity post‐reconstitution remain limited, highlighting the need for more robust and scalable storage methods—particularly for global vaccine deployment in resource‐limited settings.

Despite advances in EV functionalization, achieving targeted delivery to lymphoid or tumor tissues remains a major engineering challenge. As mentioned, native EVs exhibit a propensity to accumulate in clearance organs such as the liver and spleen, thereby limiting their immunological efficacy. To overcome this limitation and enhance biodistribution, various surface modification strategies—such as conjugation of targeting ligands, PEGylation, or CD47 overexpression—have been actively investigated.^[^ ^302^ ^]^ However, these modifications may introduce new immunological liabilities or alter EV uptake by APC. Beyond surface engineering, another critical determinant of EV behavior in vivo is their dynamic remodeling upon systemic administration.^[^ ^294^ ^]^ One prominent aspect of this remodeling is the formation of a protein corona, in which circulating serum proteins adsorb onto the EV surface and modify its physicochemical and biological properties.^[^ ^303^ ^]^ This process can obscure functional moieties such as targeting ligands, ultimately compromising receptor‐specific binding and delivery efficiency. In fact, protein corona formation has been shown to attenuate antigen presentation and impair the delivery of nucleic acid payloads, thereby reducing the immunostimulatory potential of engineered EVs. Importantly, this type of biological reconditioning is further influenced by factors introduced during EV isolation and purification, which may alter vesicle composition or surface integrity.^[^ ^304^ ^]^ Together, these challenges underscore the necessity of evaluating EV behavior under physiologically relevant conditions—considering not only their engineered features but also how they are dynamically reshaped by the host environment. Accordingly, the success of EV‐based vaccine strategies depends not only on rational design and surface functionalization but also on a systems‐level understanding of their fate and function post‐administration. Therefore, striking a balance between prolonged circulation and effective immune engagement remains a central challenge in EV vaccine engineering.

Antigen loading strategies similarly exhibit trade‐offs. Exogenous loading methods such as electroporation and sonication offer versatility in incorporating a wide range of cargoes, but may compromise vesicle integrity. In contrast, endogenous loading through donor cell engineering leverages native exosomal biogenesis pathways to enable physiological cargo incorporation, though this approach may be limited by suboptimal antigen loading efficiency and the potential to induce unintended immune activation.^[^ ^305^ ^]^ Targeting specificity, loading efficiency, and immune evasion are interrelated parameters that require integrated optimization. Current bioengineering strategies provide partial solutions but may also introduce new complications that warrant careful evaluation. Therefore, comparative studies are essential to identify the approach that elicits the most consistent and therapeutically relevant immune responses across diverse disease models.

The therapeutic efficacy of EV‐based vaccines is increasingly determined by how strategically they are integrated into combination therapies. ICB agents, particularly anti‐CTLA‐4 or anti‐PD‐1 antibodies, have emerged as potent partners in this regard, synergizing with EV‐based platforms to amplify T cell responses. In a representative example, Phung et al. developed a hybrid system wherein OVA‐pulsed DEX were surface‐functionalized with anti‐CTLA‐4 antibodies via post‐insertion of DPPE‐PEG–linked immunoglobulins.^[^ ^125^ ^]^ This design significantly enhanced antigen‐specific T cell priming in tumor‐draining lymph nodes, demonstrating how combinatorial membrane engineering can augment endogenous MHC presentation. Beyond molecular conjugation, physical stimuli such as hyperthermia or irradiation have been employed to endow EVs with intrinsic immunogenicity. Liu et al. encapsulated black phosphorus quantum dots (BPQDs) within EVs derived from hyperthermia‐treated tumor‐bearing mice—whose thermal stress increases tumor antigen content and enhances immunogenicity—creating a photo‐nanovaccine (hEX@BP) that achieves synergistic therapeutic efficacy via near‐infrared‐induced photothermal tumor ablation immunogenic T cell activation through DC maturation.^[^ ^113^ ^]^ Extending this conceptual framework, recent studies have incorporated EVs into biomaterial platforms such as chemically crosslinked hydrogels, in which EVs are repurposed as structural building blocks within bioorthogonally crosslinked PEG matrices to support localized immune activation and sustained vesicle retention.^[^ ^306^ ^]^ These systems exemplify a paradigm shift from EVs as passive delivery vehicles to dynamic, immunoactive components of therapeutic scaffolds. Remarkably, EV‐loaded protein hydrogels have been shown to induce the de novo formation of tumor‐localized tertiary lymphoid structures, even in the absence of genetic modification, using only spleen‐derived EVs.^[^ ^307^ ^]^ This approach transcends conventional immune activation by inducing spatially organized immune microenvironments—characterized by CXCL13^+^ stromal networks and PNAd^+^ high endothelial venules—that support structured and sustained antitumor immunity. In immunologically cold tumor models, the EV‐based hydrogel synergized with ICB therapy to convert nonresponsive tumors into inflamed, lymphocyte‐infiltrated environments. Collectively, these approaches highlight a paradigm shift in which EV‐based vaccines function not merely as passive antigen carriers but as dynamically engineered immunotherapeutic systems. Through strategic combinations with ICB, incorporation of physical or biomaterial cues, EV platforms are increasingly being tailored to reshape immune responses, enhance antigen specificity, and overcome resistance across diverse tumor microenvironments.

## 5 Conclusion

EV‐based vaccines have emerged as a promising next‐generation immunotherapeutic modality, distinguished by their inherent biocompatibility, intrinsic immunomodulatory potential, and exceptional versatility for bioengineering. Early‐phase clinical studies—particularly those employing DEXs—have demonstrated preliminary safety and feasibility, affirming the translational potential of these acellular vesicles.^[^ ^86^, ^134^, ^135^, ^197^ ^]^ Expanding beyond DEXs, the growing repertoire of TEXs and immune cell‐derived EVs—including those from macrophages, T cells, B cells, NK cells, and neutrophils—has further broadened the therapeutic landscape by revealing distinct molecular signatures and immunological functionalities tailored to diverse clinical needs.

Throughout this work, we examined a broad spectrum of antigen loading strategies, ranging from exogenous antigen incorporation techniques such as electroporation and sonication to endogenous approaches utilizing donor cell engineering. Each method presents unique strengths and limitations in terms of antigen density, stability, and immune accessibility, underscoring the need for context‐dependent optimization. To fully realize the therapeutic potential of these design strategies, however, they must be integrated with appropriate source cell selection tailored for targeted immunological responses, robust and standardized cargo loading methods that ensure high reproducibility and quality control, and delivery strategies optimized for precise biodistribution and uptake. This multifactorial design paradigm necessitates coordinated advances across biological and technological domains to ensure translational success. Addressing persistent challenges—including scalable production, batch‐to‐batch reproducibility, and regulatory standardization—will be essential for translating these complex platforms into clinically viable therapies.

Looking forward, continued progress in nanotechnology and immunoengineering will enable more precise control over EV cargo composition, context‐dependent immune activation, and organ‐ or tissue‐specific targeting. Coupled with well‐designed combination strategies such as ICB and hydrogel‐based delivery systems, EV‐based vaccines are poised to reshape the future of immunomodulation across cancer, infectious diseases, and chronic inflammatory conditions.

## Conflict of Interest

The authors declare no conflict of interest.

## Author Contributions

**Wonkyung Ahn**: conceptualization (lead); visualization (supporting); writing—original draft (lead). **Yeram Lee**: conceptualization (lead); visualization (lead); writing—original draft (supporting). **Gi‐Hoon Nam**: writing—review & editing (supporting). **Jae Bem You**: writing—review & editing (supporting). **Eun Jung Lee**: supervision (lead); writing—review & editing (lead). **Wonkyung Ahn** and **Yeram Lee** contributed equally to this work.
